# Incidence and risk of hypertension associated with PARP inhibitors in cancer patients: a systematic review and meta-analysis

**DOI:** 10.1186/s12885-023-10571-5

**Published:** 2023-01-31

**Authors:** Xiu Chen, Qinglian Wen, Liqiu Kou, Xiaolu Xie, Jun Li, Yaling Li

**Affiliations:** 1 Department of Pharmacy, The Affiliated Hospital of Southwest Medical University, Luzhou, China; 2 School of Pharmacy, Southwest Medical University, Luzhou, China; 3 Department of Oncology, The Affiliated Hospital of Southwest Medical University, Luzhou, China; 4 Department of Traditional Chinese Medicine, The Affiliated Hospital of Southwest Medical University, Luzhou, China

**Keywords:** PARP inhibitors, Hypertension, Niraparib, Olaparib, Meta-analysis

## Abstract

**Objective:**

To analyze the incidence and risk of hypertension associated with poly(adenosine diphosphate-ribose) polymerase (PARP) inhibitors in cancer patients and provide reference for clinicians.

**Methods:**

We used R software to conduct a meta-analysis of phase II/III randomized controlled trials (RCT) on PARP inhibitors for cancer treatment published in PubMed, Embase, Clinical Trials, Cochrane Library and Web of Science from inception to July 29th, 2022.

**Results:**

We included 32 RCTs with 10,654 participants for this meta-analysis. For total PARP inhibitors, the incidence and risk ratio of all-grade hypertension were 12% and 1.22 (95% CI: 0.91–1.65, *P* = 0.19, I^2^ = 81%), and the incidence and risk ratio of grade 3–4 hypertension were 4% and 1.24 (95% CI: 0.74–2.08, *P* = 0.42, I^2^ = 68%). Compared with the control group, the niraparib group, olaparib 800 mg/day group, and olaparib plus cediranib group increased the risk of any grade and grade 3–4 hypertension, while the veliparib group and rucaparib group did not increase the risk of any grade and grade 3–4 hypertension, and olaparib 200 mg-600 mg/day group (exclude olaparib plus cediranib regime) reduced the risk of any grade and grade 3–4 hypertension.

**Conclusion:**

Olaparib 200-600 mg/day (excluding olaparib plus cediranib regimen) may be the most suitable PARP inhibitor for cancer patients with high risk of hypertension, followed by veliparib and rucaparib. Niraparib, olaparib 800 mg/day and olaparib combined with cediranib may increase the risk of developing hypertension in cancer patients, clinicians should strengthen the monitoring of blood pressure in cancer patients and give medication in severe cases.

**Supplementary Information:**

The online version contains supplementary material available at 10.1186/s12885-023-10571-5.

## Introduction

Cancer is a serious threat to human health, causing more than 8 million deaths each year [1]. Targeted therapy with high efficiency and low toxicity is the main strategy for the treatment of advanced cancer, which can specifically kill cancer cells with minimal harm to normal cells. For targeted therapy of cancer, it is of great significance to identify new drug targets and develop new targeted drugs [2]. DNA damage response (DDR) is a complex signal pathway network involving DNA damage repair, cell cycle checkpoint and apoptosis, which has become an important target in the development of new targeted therapeutic drugs [3]. In the past few years, DNA damage response and its related signal pathways have attracted considerable attention, and a large number of DDR inhibitors have emerged, such as PARP inhibitors, ataxia telangiectasia-mutated (ATM) inhibitors, ataxia telangiectasia and Rad3-related (ATR) kinase inhibitors and checkpoint kinase 1/2 (CHK1/2) inhibitors, etc. [4].

PARP inhibitors are currently the most widely studied DDR inhibitors, which can cause simultaneous impairment of two different DDR pathways (homologous recombination and base excision repair) by inhibiting the PARP protein, leading to apoptotic death of cancer cells through a mechanism known as "synthetic lethality" [5]. The PARP inhibitors developed so far include veliparib, rucaparib, olaparib, talazoparib, niraparib, pamiparib, iniparib, fuzuloparib etc. Surprisingly, It has been found that PARP inhibitors alone or in combination (e.g. platinum drugs) show promising clinical efficacy in various cancer patients, especially those with impaired homologous recombination [6, 7]. From 2014 to August 25, 2022, olaparib, rucaparib, talazoparib and niraparib have been clinically approved by FDA and/or the European Medicines Agency (EMA) for the treatment of various cancers (e.g. ovarian cancer, breast cancer, lung cancer) [8–11]. Niraparib has even been approved for the first-line maintenance treatment of platinum-responsive advanced ovarian cancer, and olaparib has been approved for the first-line maintenance treatment of advanced ovarian cancer with BRCA mutation and metastatic pancreatic cancer with gBRCA mutation [12–14]. In addition, fuzuloparib and pamiparib have recently been approved for ovarian, fallopian tube or primary peritoneal cancer in china [15, 16].

PARP inhibitors, like other targeted therapeutic, are associated with many adverse reactions, among which nausea, vomiting, fatigue, anemia, thrombocytopenia, neutropenia and hypertension are frequently reported. Interestingly, the reported incidence of PARP-related hypertension in clinical trials varies widely, ranging from approximately 1% to 76%, and the reported severity also varies greatly, ranging from grade 1 to grade 4, even serious hypertension. The reasons for the above differences are unclear, and it is also unclear whether there are differences among different PARP inhibitors, different cancer types and different treatment regimes. Hypertension is the leading cause of attributable deaths and burden of disease globally, which is also one of the important preventable risk factors for cardiovascular disease [17]. For clinicians, it is necessary to have a deep understanding of PARP inhibitor-related hypertension in cancer patients, so as to minimize the risk and harm of PARP inhibitor-related hypertension and ensure the maximum benefit of cancer patients. Given this background, we conducted a comprehensive meta-analysis of published Phase II and III RCTs of PARP inhibitors in the treatment of cancer to determine the incidence and risk of PARP inhibitors and to analyze the differences in the risk of hypertension among different PARP inhibitors, different cancer types and different treatment regimens. We hope to provide reference for clinicians to reasonably use PARP inhibitors and manage hypertension related to PARP inhibitors.

## Methods

This study followed the Preferred Reporting Items for Systematic reviews and Meta-Analyses (PRISMA) guidelines.

### Literature search

We searched PubMed, Embase, Cochrane Library, Web of Science, and ClinicalTrials.gov databases to identify relevant II/III randomised controlled trials published from inception to July 29, 2022, without language restrictions. We searched for the following keywords: veliparib, rucaparib, olaparib, talazoparib, niraparib, pamiparib, iniparib, fuzuloparib, PARP inhibitor, and used the RCTs filter or searched for randomly or randomized or randomization or random in the full text to identify possible RCTs. In addition, we have reviewed the references of the retrieved literature to identify any possible relevant studies.

### Selection criteria

We searched for phase II or III RCTs of PARP inhibitors in the treatment of cancer patients. Inclusion criteria were based on the PICO-framework. Population (P): cancer patient. Intervention (I): Treatments containing PARP inhibitors. Comparison (C): Placebo or treatments without PARP inhibitors. Outcomes (O): any grade hypertension and grade 3–4 hypertension assessed according to the National Cancer Institute's Common Terminology Standard for Adverse Events (CTCAE) (version 3 or 4).

The exclusion criteria were as follows: (a) non-randomized controlled trials; (b) review and guideline; (c) trails with unavailable study data; (d) investigation; (e) conference articles; (f) both arms contain PARP inhibitors; (f) Phase I study. When there is a dispute between two reviewers, the decision is made by the third reviewer (YL).

### Data extraction

We extracted data from articles, supplementary documents and ClinicalTrials.gov. Two reviewers (XC and XX) independently extracted the following information: author/year, national clinical trial (NCT) number, nation, study phase, interventions, sample size, median age, median treatment duration, median follow up duration and cancer type.

### Quality assessment

According to the Cochrane Collaboration guidelines, we assessed the risk of bias for included RCTs from seven domains: random sequence generation, allocation concealment, blinding of participants and personnel, blinding of outcome assessment, incomplete outcome data, selective reporting, and other types. The evaluation results are low, high and unclear, indicating low risk of bias, high risk of bias and unclear risk of bias, respectively.

### Statistical analysis

All statistical analyses in our meta-analysis were performed by R software(version 4.0.2). To calculate the incidence of any grade hypertension and grade 3–4 hypertension, we determined the number of patients with any grade hypertension and grade 3–4 hypertension in patients receiving PARP inhibitors alone or in combination in each study and the total number of patients receiving PARP inhibitors alone or in combination. Freeman-Tukey double arcsine transformation was used to stabilize the variance when calculating the proportion of patients and 95% confidence intervals (CIs). Analyzing the risk of any grade hypertension and the risk of grade 3–4 hypertension associated with PARP inhibitors in cancer patients is our second objective. Risk ratio (RR) and 95% CI were used to determine the risk of hypertension with PARP inhibitors group compared to control group. Both random-effects(Mantel–Haenszel method) and fixed-effects models(Mantel–Haenszel method) were used to draw forest plots. We used Cochran 's Q test to assess heterogeneity among studies and the inconsistency index (I^2^ test) to assess the degree of heterogeneity. If there was no statistical heterogeneity among the studies (I^2^ < 50%), the fixed effects model was used for analysis; otherwise, the random effect model was used for analysis. Study exclusions and subgroup analyses were used to identify the main sources of heterogeneity. Publication bias was evaluated by visual inspection of funnel plots and Begg's tests. *P* < 0.05 was statistically significant.

## Results

### Selection of Eligible Studies

**Fig. 1:**
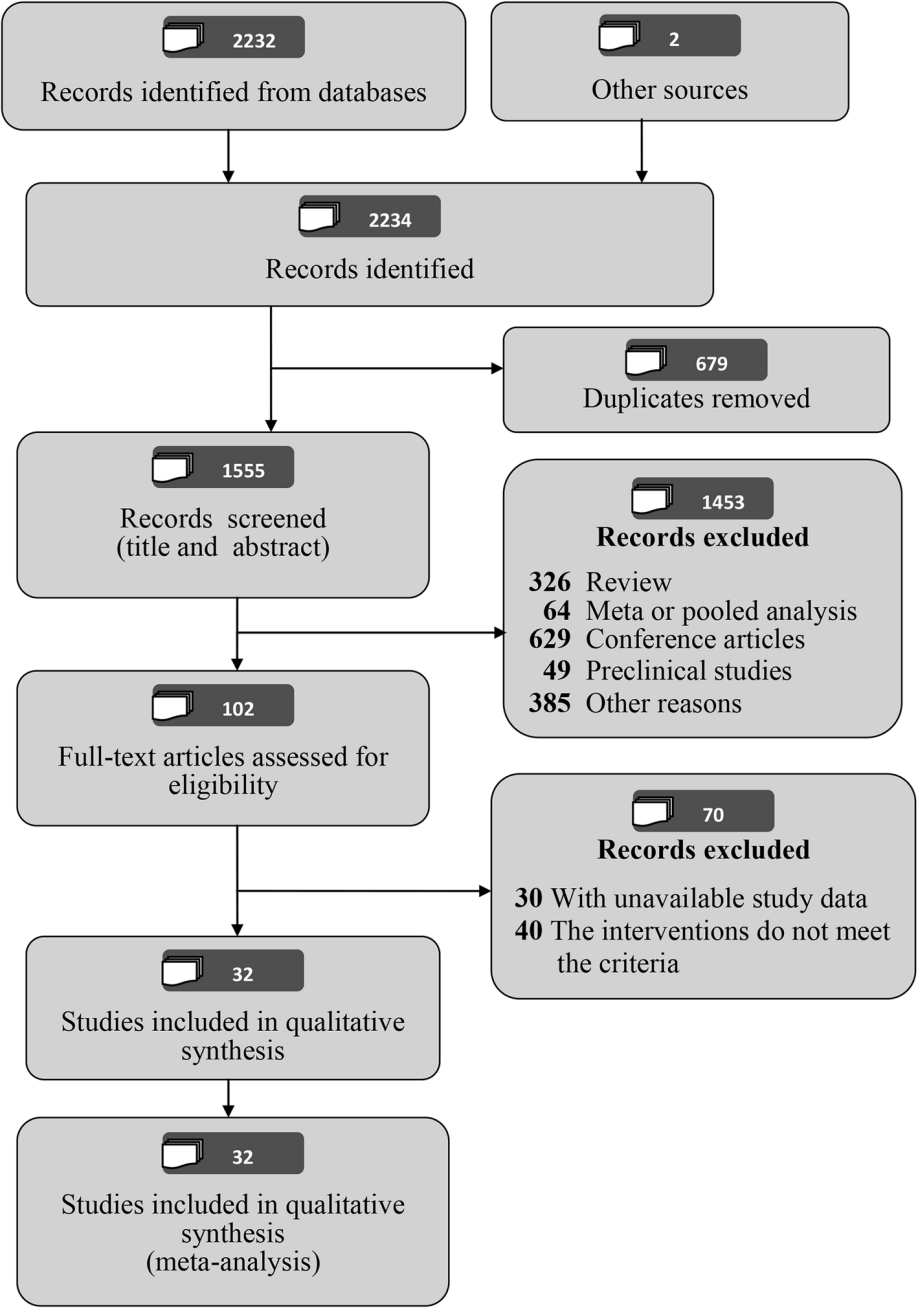
The PRISMA flowchart shows the selection process of the systematic review

According to the search strategy, a total of 2234 articles were identified. First, we removed 679 duplicate articles with EndNote software. Then, we excluded 1453 articles after screening the title and abstract, and excluded 70 articles after reading the full text. Ultimately, 32 articles [18–49] were eligible for analysis. Figure 1 shows a flow chart depicting the articles selection process.

### Characteristics of Eligible Studies

This meta-analysis included 10,654 patients with ovarian, lung, breast and other cancers from 16 phase II [18–26, 31, 35, 41, 43, 47–49] studies and 16 phase III studies [27–30, 32–34, 36–40, 42, 44–46]. 6631 participants from the PARP inhibitor group received five PARP inhibitors niraparib (*N* = 4), olaparib (*N* = 14), veliparib (*N* = 10), rucaparib (*N* = 3),and iniparib (*N* = 1), alone or in combination with other anticancer drugs, and 4023 participants from the control group received placebo, paclitaxel, carboplatin, gemcitabine, and other anticancer drugs. The median duration of treatment with PARP inhibitors reported in the included studies ranged from 44 days to 14.7 months. The characteristics of the included studies are shown in Tables 1 and 2.

**Table 1:** Characteristics of the RCTs and patients included in the meta-analysis

**Number**	**Author/year**	**NCT Number**	**Nation**	**Study phase**	**Interventions**		**Sample size**		**Median age (P/C) years**	**Median treatment duration (P/C)**	**Cancer type**
					**PARP group**	**Control group**	**PARP inhibitor group**	**Control group**			
1	Kaye [18] et.al 2011	NCT00628251	United States, Australia, etc	II	Olaparib (400 mg/day)	Liposomal doxorubicin	32	32	58.5/53	NA	Ovarian cancer
					Olaparib (800 mg/day)		32		53.5/53	NA	Ovarian cancer
2	Ledermann [19] et.al 2012	NCT00753545	United States, Austria, etc	II	Olaparib (800 mg/day)	Placebo	136	128	58/59	206.5/141 days	Ovarian cancer
3	Pusztai [20] et.al 2021	NCT01042379	United States	II	Olaparib (200 mg/day) + Durvalumab + Paclitaxel + Doxorubicin + Cyclophosphamide	Paclitaxel + Doxorubicin + Cyclophosphamide	73	299	46/48	NA	Breast cancer
4	Rugo [21] et.al 2016			II	Veliparib (100 mg/day) + Carboplatin + Paclitaxel	Carboplatin + Paclitaxel	72	44	48.5/47.5	182/165 days	Breast Cancer
5	Novello [22] et.al 2014	NCT01086254	France, Germany, etc	II	Iniparib (11.2 mg/kg/week) + Gemcitabine + Cisplatin	Gemcitabine + Cisplatin	78	39	59/58	15/13.9 weeks	Lung cancer
6	Han [23] et.al 2018	NCT01506609	United States, Australia, etc	II	Veliparib (240 mg/day) + Carboplatin + Paclitaxel	Placebo + Carboplatin + Paclitaxel	93	96	44/46	36/30 weeks	Breast Cancer
					Veliparib (80 mg/day) + Temozolomide		93		46/46	18/30 weeks	Breast Cancer
7	Hussain [24] et.al 2017	NCT01576172	United States	II	Veliparib (600 mg/day) + Abiraterone acetate + Prednisone	Abiraterone acetate + Prednisone	79	74	68/69	36/36 weeks	Prostate Cancer
8	Owonikoko [25] et.al 2020	NCT01642251	United States	II	Veliparib (200 mg/day) + Etoposide + Cisplatin	Placebo + Etoposide + Cisplatin	66	66	66/64	NA	Lung Cancer
9	Fennell [26] et.al 2022	NCT01788332	United Kingdom	II	Olaparib (600 mg/day)	Placebo	31	38	65/63	12/12 weeks	Lung cancer
10	Banerjee [27] et.al 2021	NCT01844986	United States, Canada, etc	III	Olaparib (600 mg/day)	Placebo	260	130	NA	24.6/13.9 months	Ovarian cancer
11	Mirza [28] et.al 2016	NCT01847274	United States, Italy, etc	III	Niraparib (300 mg/day)	Placebo	367	179	NA	NA	Ovarian cancer
12	Bang [29] et.al 2017	NCT01924533	China, Japan, etc	III	Olaparib (200 mg/day) + Paclitaxel	Placebo + Paclitaxel	262	259	58/59	73.5/59 days	Gastric cancer
13	Ledermann [30] et.al 2021	NCT01968213	United States, Australia, etc	III	Rucaparib (1200 mg/day)	Placebo	372	189	61/62	8.3/5.5 months	Ovarian cancer
14	Clarke [31] et.al 2018	NCT01972217	United States, Canada, etc	II	Olaparib (600 mg/day) + Abiraterone	Pacebo + Abiraterone	71	71	70/67	309/253 days	Prostate cancer
15	Loibl [32] et.al 2018	NCT02032277	United States, Australia, etc	III	Veliparib (100 mg/day) + Paclitaxe + Carboplatin	Veliparib placebo + Paclitaxel + Carboplatin	313	158	51/49	89/85.5 days	Breast cancer
						Veliparib placebo + Paclitaxel + Carboplatin placebo		157	51/50	89/84 days	Breast cancer
16	Diéras [33] et.al.2020	NCT02163694	United States, Australia, etc	III	Veliparib (240 mg/day) + Carboplatin + Paclitaxel	Placebo + Carboplatin + Paclitaxel	336	171	47/45	NA	Breast cancer
17	Golan [34] et.al 2020	NCT02184195	United States, Canada, etc	III	Olaparib (600 mg/day)	Placebo	90	61	57/57	6/3.7 months	Pancreatic Cancer
18	Gorbunova [35] et.al 2019	NCT02305758	North America, Australia etc	II	Veliparib (400 mg/day) + Irinotecan + Leucovorin + Fluorouracil infusion ± Bevacizumab	Irinotecan + Leucovorin + Fluorouracil bolus + Fluorouracil infusion ± Bevacizumab	65	65	59/64	NA	Colorectal Cancer
19	Liu [36] et.al 2022	NCT02446600	United States, Canada, Japan	III	Olaparib (600 mg/day)	Platinum-based chemotherapy	187	167	NA	NA	Ovarian, Fallopian Tube, or Primary Peritoneal Cancer
					Olaparib (400 mg/day) + Cediranib		183		NA	NA	Ovarian, Fallopian Tube, or Primary Peritoneal Cancer
20	Coleman [37] et.al 2019	NCT02470585	United States, Australia, etc	III	Veliparib (300 mg/day) + Carboplatin + Paclitaxel + followed by veliparib (600 mg or 800 mg/day) maintenance	Placebo + Carboplatin + Paclitaxel + followed by Placebo maintenance	377	371	62/62	NA	Ovarian, Fallopian Tube, or Primary Peritoneal Cancer
					Veliparib (300 mg/day) + Carboplatin + Paclitaxel + followed by Placebo maintenance		376		62/62	NA	Ovarian, Fallopian Tube, or Primary Peritoneal Cancer
21	Ray-Coquard [38] et.al 2019	NCT02477644	Austria, Belgium, etc	III	Olaparib (600 mg/day) + Bevacizumab	Placebo + Bevacizumab	535	267	61/60	17.3/15.6 moths	Ovarian cancer
22	González-Martín [39] et.al 2019	NCT02655016	United States, France, etc	III	Niraparib (200 mg or 300 mg/day)	Placebo	484	244	62/62	NA	Ovarian cancer
23	Kristeleit [40] et.al 2022	NCT02855944	United States, Brazil, etc	III	Rucaparib (1200 mg/day)	Chemotherapy (administered per institutional guidelines)	232	113	58/59	7.3/3.6 months	Ovarian cancer
24	Chiorean [41] et.al 2021	NCT02890355	United States	II	Veliparib (400 mg/day) + Irinotecan + Folinic acid + 5-Fluorouracil infusion	Irinotecan + Folinic acid + 5-Fluorouracil bolus + 5-Fluorouracil infusion	56	50	67/67	8/10 weeks	Pancreatic Cancer
25	Bono [42] et.al 2020	NCT02987543	United States, Canada, etc	III	Olaparib (600 mg/day)	Physician’s choice of enzalutamide or abiraterone	256	130	NA	7.4/3.9 months	Prostate Cancer
26	Colombo [43] et.al 2022	NCT03314740	Italy	II	Olaparib (600 mg/day) every day + Cediranib every day	Paclitaxel	41	28	64.2/62.5	NA	Ovarian cancer
					Olaparib (600 mg/day) every day + Cediranib 5 days a week		41		59.9/62.5	NA	Ovarian cancer
27	Ai [44] et.al 2021	NCT03516084	China	III	Niraparib (300 or 200 mg/day)	Placebo	125	60	NA	44/42.5 days	Lung Cancer
28	Monk [45] et.al 2022	NCT03522246	United States, Australia, etc	III	Rucaparib (1200 mg/day)	Placebo	425	110	61/61	14.7/9.9 month	Ovarian cancer
29	Wu [46] et.al 2020	NCT03705156	China	III	Niraparib (300 mg or 200 mg/day)	Placebo	177	88	53/55	369/171 days	Ovarian cancer
30	Woll [47] et.al 2022	/	United Kingdom	II	Olaparib (300 mg twice a day)	Placebo	73	74	66/64	8/8 weeks	Lung cancer
					Olaparib ( 200 mg three times a day)	Placebo	73		63/64	19/8 weeks	Lung cancer
31	Sun [48] et.al 2022	/	China	II	Olaparib (400 mg/day) + Bevacizumab	Albumin-bound paclitaxel + Bevacizumab	42	42	NA	NA	Ovarian cancer
32	O’Reilly [49] et.al 2020	/	United States, Canada, Israel	II	Veliparib (160 mg/day) + Cisplatin + Gemcitabine	Cisplatin + Gemcitabine	27	23	64/63	NA	Pancreas Adenocarcinoma

*NCT number* national clinical trial number, *PARP* poly(adenosine diphosphate-ribose) polymerase, *NA* not reported / Not registered on the ClinicalTrials.gov, *P/C* PARP inhibitor group/control group

**Table 2:** Summary of included RCTs

**PARP Inhibitors**	**Number of Phase II Studies**	**Number of Phase III Studies**	**Sample Size (PARP group/control group)**	**Interventions**		**Median treatment duration (PARP group)**	**Cancer**
				**Treatment regime (PARP group versus control group)**	**Number of studies**		
Niraparib	0	4	1153/571	A	4	44 days, 369 days	Ovarian cancer, Lung Cancer
Olaparib	8	6	2418/1726	A	5	8 weeks ~ 24.6 months	Ovarian cancer, lung cancer, pancreatic cancer, prostate cancer, breast cancer, and gastric cancer
				B	3		
				C	6		
Veliparib	7	3	1953/1275	C	10	8 weeks ~ 36 weeks	Ovarian cancer, breast cancer, prostate cancer, lung cancer, colorectal cancer, pancreatic cancer, pancreas adenocarcinoma
Rucaparib	0	3	1029/412	A	2	7.3 months ~ 14.7 months	Ovarian cancer, peritoneal cancer
				B	1		
Iniparib	1	0	78/39	C	1	15 weeks	Lung cancer
Totle	16	16	6631/4023	A,B,C	32	44 days ~ 14.7 months	Ovarian cancer, lung cancer, pancreatic cancer, prostate cancer, breast cancer, peritoneal cancer, colorectal cancer, gastric cancer, pancreas adenocarcinoma

*PARP* poly(adenosine diphosphate-ribose) polymerase, *A*: PARP inhibitors versus placebo, *B*: PARP inhibitors versus other anticancer drugs, *C*: PARP inhibitors + other anticancer drugs versus other anticancer drugs

### Evaluation of the quality of RCTs

We assessed the quality of the 32 included double-blind randomized controlled trials [18–49] according to the Cochrane Collaboration guidelines. 11 [18, 20–22, 24, 36, 40–43, 49] of the 32 included studies were open-label studies and were not blinded. Of the remaining 21 studies, 2 studies [33, 37] mentioned that outcome assessors were not blinded, and one study [37] mentioned that drug allocation concealment was not performed. Most RCTs were conducted strictly according to the Cochrane Collaboration guidelines, and the overall quality was high. See Supplementary Table 1 for details.

### Incidence of hypertension associated with PARP inhibitors

**Fig. 2:**
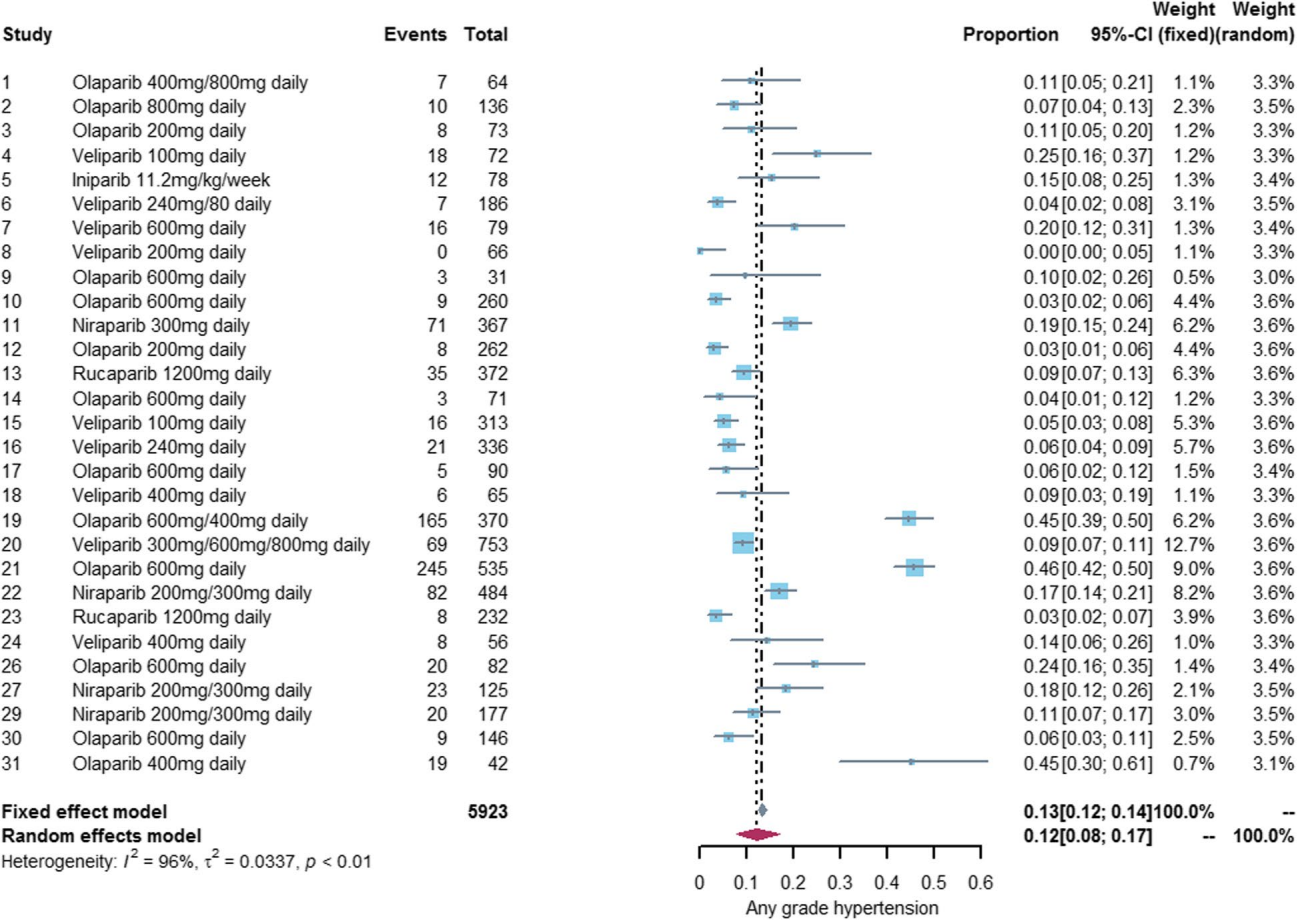
Forest plot of incidence of any grade hypertension related to PARP inhibitor

**Fig. 3:**
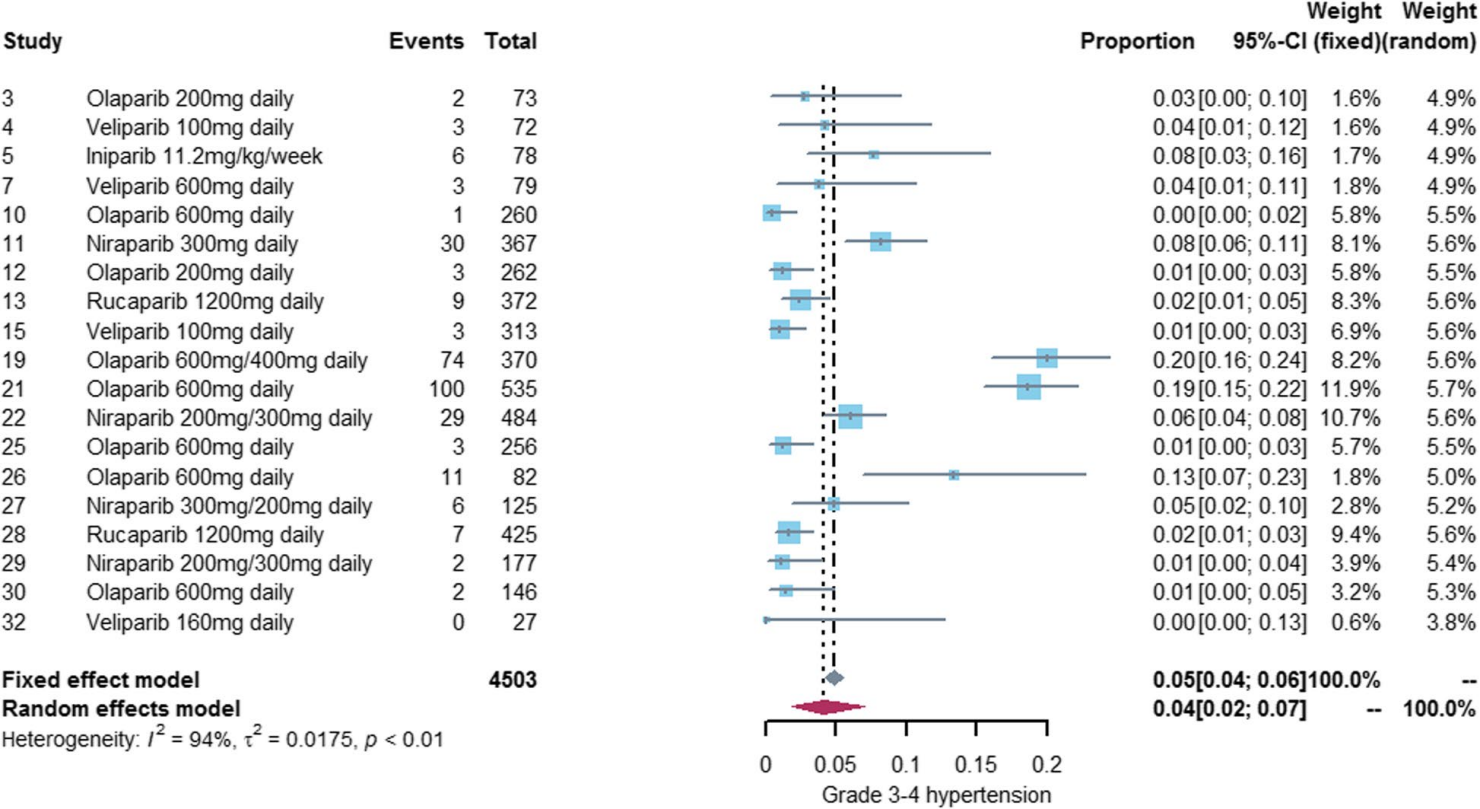
Forest plot of incidence of grade 3-4 hypertension related to PARP inhibitor

**Fig. 4:**
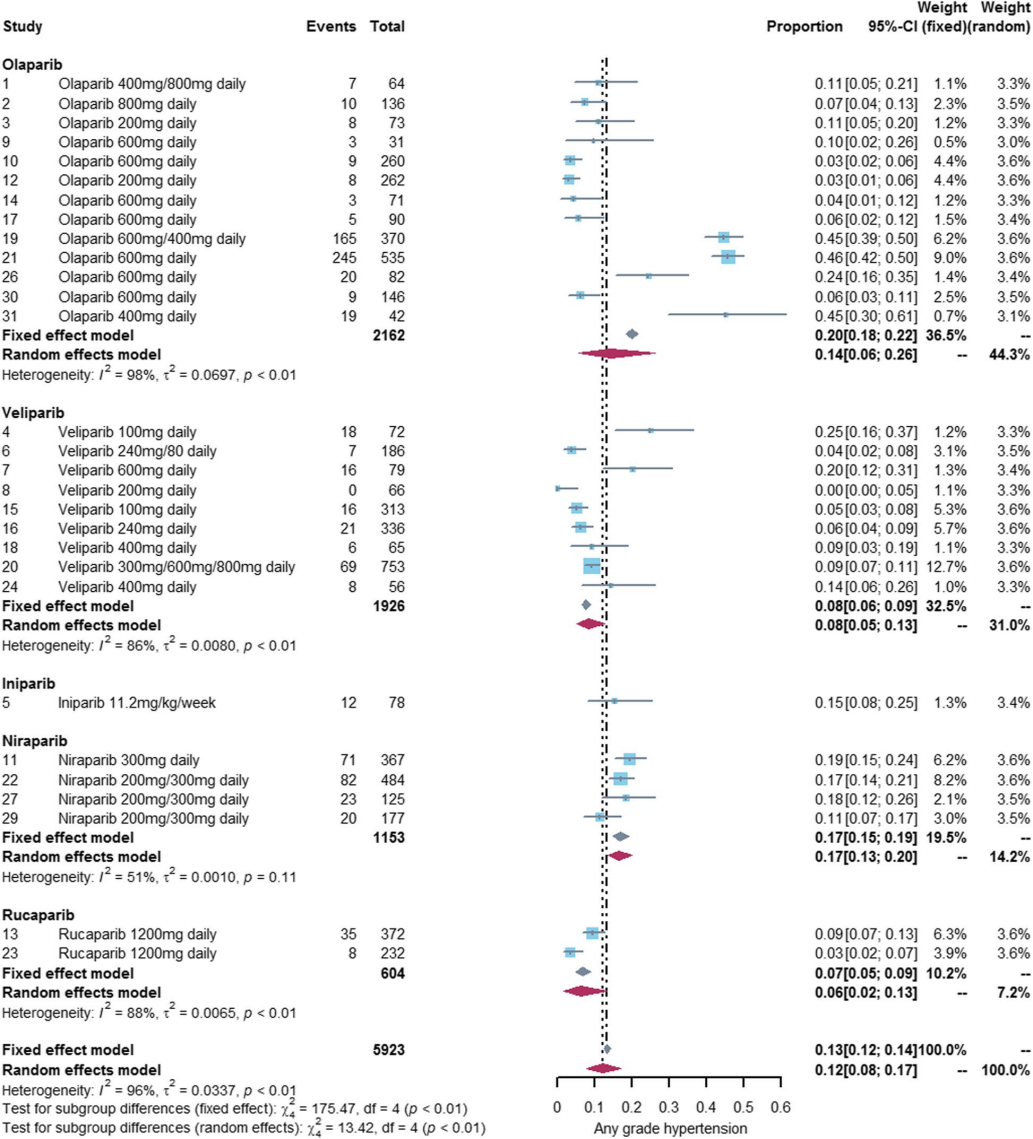
Forest plot of incidence of any grade hypertension related to different PARP inhibitors

**Fig. 5:**
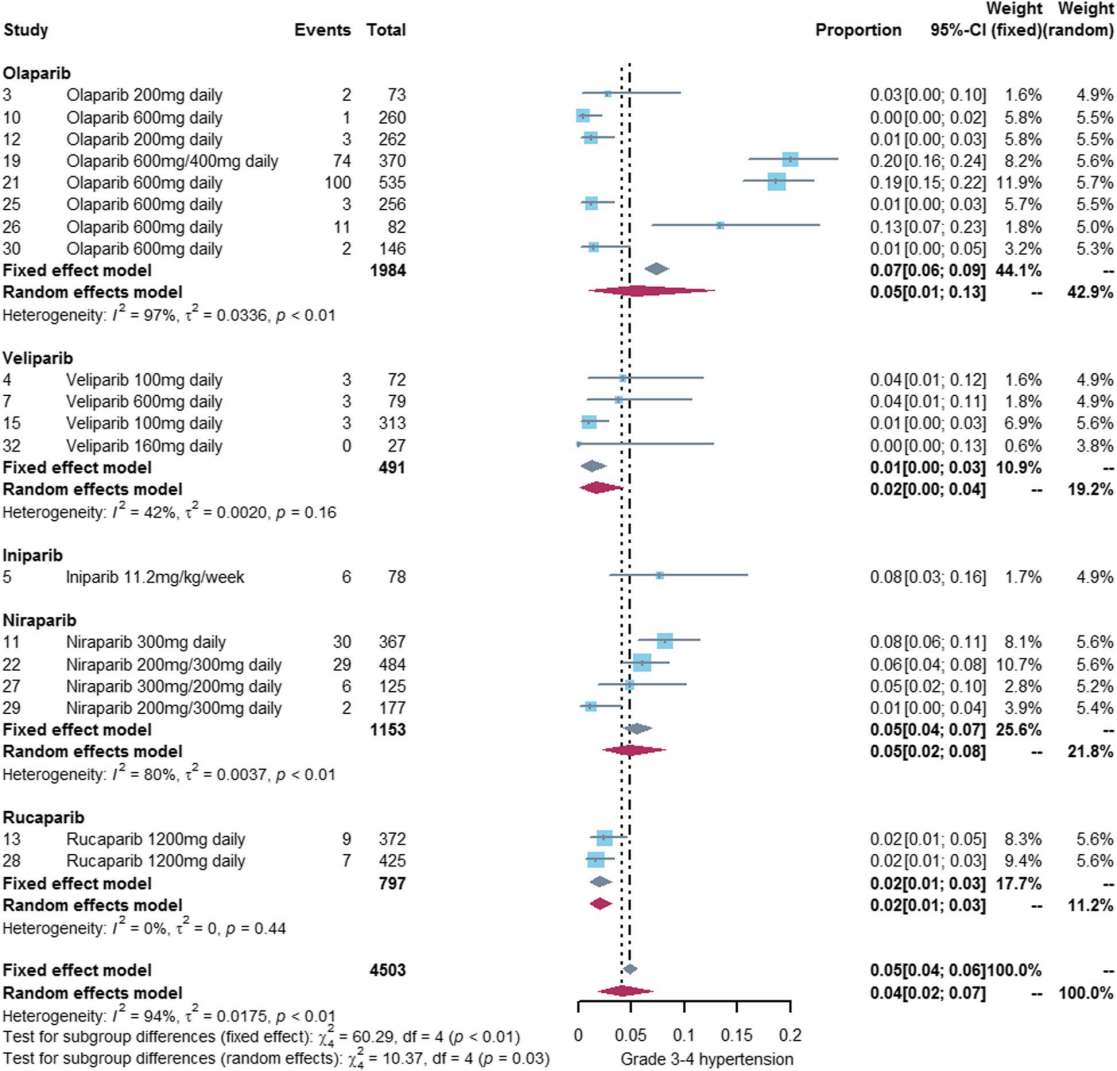
Forest plot of incidence of grade 3-4 hypertension related to different PARP inhibitors

We performed a meta-analysis of 29 studies [18–41, 43, 44, 46–48] reporting any grade hypertension and 19 studies [20–22, 24, 27–30, 32, 36, 38, 39, 42–47, 49] reporting grade3-4 hypertension. The incidence of any grade hypertension was 12% (95%CI: 8%-17%) and the incidence of grade 3–4 hypertension was 4% (95%CI: 2%-7%). See Figs. 2 and 3 for details. The incidence of hypertension varies widely among different PARP inhibitors, with olaparib (any grade:14%, grade3-4: 5%) and niraparib (any grade:17%, grade3-4: 5%) exhibiting a higher incidence of hypertension than veliparib (any grade:8%, grade3-4: 1%) and rucaparib (any grade:6%, grade3-4: 2%). Only one study [22] reported hypertension associated with iniparib, so we did not conduct meta-analysis of iniparib alone. See Figs. 4 and 5 for details.

### Risk of Hypertension Associated with PARP Inhibitors

We performed a meta-analysis of 29 studies [18–41, 43, 44, 46–48] reporting hypertension of any grade and 19 studies [20–22, 24, 27–30, 32, 36, 38, 39, 42–47, 49] reporting grade 3–4 hypertension, respectively. There was considerable heterogeneity among studies, so we used a random-effects model for analysis. There was no statistically significant difference in the risk of hypertension between the PARP inhibitor group and the control group(any grade: RR = 1.22, 95% CI: 0.91–1.65, *P* = 0.19, I^2^ = 81%; grade3-4: RR = 1.24, 95% CI: 0.74–2.08, *P* = 0.42, I^2^ = 68%). See Table 3 and Supplementary Fig. 1 for details.

### Subgroup analysis of hypertension risk

We conducted subgroup analysis to explore the difference of hypertension risk among different PARP inhibitors, different cancer types and different treatment regimes.

#### Subgroup analysis of PARP inhibitors

Our subgroup analysis showed that the risk of hypertension varied widely among different PARP inhibitors. The risk of hypertension was significantly higher in the niraparib group compared with the control group (any grade: RR = 3.47, 95% CI: 2.36–5.09, *P* < 0.01, I^2^ = 21%; grade3-4: RR = 4.20, 95% CI: 2.04–8.68, *P* < 0.01, I^2^ = 0%). However, veliparib (any grade: RR = 1.01, 95% CI: 0.80–1.28, *P* = 0.94, I^2^ = 3%; grade 3–4: RR = 0.77, 95% CI: 0.32–1.83, *P* = 0.55, I^2^ = 0%) and rucaparib (any grade: RR = 0.90, 95% CI: 0.56–1.45, *P* = 0.67, I^2^ = 16%; grade 3–4: RR = 0.77, 95% CI: 0.34–1.74, *P* = 0.53, I^2^ = 15%) showed a comparable risk of hypertension as the control group. See Table 3 and Supplementary Fig. 2 for details.

**Table 3:** Summary of the risk of hypertension associated with PARP inhibitors

Subgroup			Any grade				Grade 3–4			
			RR(95%CI)	*P*	I^2^	Model	RR(95%CI)	*P*	I^2^	model
**PARP inhibitors**										
Olaparib	Olaparib plus cediranib regimen		8.78(5.39,14.29)	< 0.01	0%	Fixed	6.50(3.50,12.05)	< 0.01	0%	Fixed
	Olaparib (exclude olaparib plus cediranib regimen)	200 mg-600 mg/day	0.79(0.69,0.89)	< 0.01	22%	Fixed	0.61(0.48,0.77)	< 0.01	0%	Fixed
		800 mg/day	2.71(1.10,6.69)	0.03	23%	Fixed	/			
		All dose	0.82(0.72,0.92)	< 0.01	38%	Fixed	0.61(0.48,0.77)	< 0.01	0%	Fixed
	Olaparib (include olaparib plus cediranib regimen)		1.12(0.67,1.87)	0.67	86%	Random	1.08(0.45,2.61)	0.86	79%	Random
Veliparib			1.01(0.80,1.28)	0.94	3%	Fixed	0.77(0.32,1.83)	0.55	0%	Fixed
Niraparib			3.47(2.36,5.09)	< 0.01	21%	Fixed	4.20(2.04,8.68)	< 0.01	0%	Fixed
Rucaparib			0.90(0.56,1.45)	0.67	16%	Fixed	0.77(0.34,1.74)	0.53	15%	Fixed
Iniparib			/				/			
**Cancer type**										
Ovarian cancer			1.68(0.98,2.86) 1.54(0.93,2.55)	0.10	91%	Random	1.59(0.68,3.69)	0.28	84%	Random
Lung cancer			0.79(0.33,1.90)	0.59	62%	Random	1.11(0.45,2.75)	0.83	37%	Fixed
Breast cancer			1.21(0.85,1.73)	0.41	23%	Fixed	1.09(0.42,2.78)	0.86	0%	Fixed
Prostate cancer			0.78(0.45,1.35)	0.38	0%	Fixed	0.70(0.23,2.10)	0.52	0%	Fixed
Pancreatic cancer			1.17(0.52,2.62)	0.71	0%	Fixed	/			
**Treatment regime**										
PARP versus Placebo			1.47(0.80,2.70)	0.22	78%	Random	1.37(0.57,3.29)	0.48	60%	Random
PARP + Other anticancer drugs versus Other anticancer drugs			1.19(0.78,1.80)	0.42	87%	Random	1.37(0.55,3.40)	0.50	84%	Random
PARP versus Other anticancer drugs			1.26(0.77,2.05)	0.35	48%	Fixed	0.40(0.16,1.00)	0.05	0%	Fixed
**Total**			1.22(0.91,1.65)	0.19	81%	Random	1.24(0.74,2.08)	0.42	68%	Random

*PARP* poly(adenosine diphosphate-ribose) polymerase, *RR* risk ratio, *CI* confidence interval, *I*^*2*^ The greater the value of I^2^, the greater the heterogeneity among studies. If I^2^ ≥ 50, the random effect model is used for analysis, otherwise the fixed effect model is used for analysis; *P P* < 0.05 indicates a statistical difference

There was great heterogeneity between olaparib group and the control group (any grade: I^2^ = 86%; grade 3–4: I^2^ = 79%), but the heterogeneity was significantly reduced when 2 studies [36, 43] of olaparib plus cediranib were excluded (any grade: I^2^ = 38%; grade3-4: I^2^ = 0%). The risk of hypertension with olaparib plus cediranib regime was significantly higher than that in the control group (any grade: RR = 8.78, 95% CI: 5.39–14.29, *P* < 0.01, I^2^ = 0%; grade 3–4: RR = 6.50, 95% CI: 3.50–12.05, *P* < 0.01, I^2^ = 0%), while the risk of hypertension with olaparib (exclude olaparib plus cediranib regime) alone or in combination with other anticancer drugs was lower than that in the control group (any grade: RR = 0.82, 95% CI: 0.72–0.92, *P* < 0.01, I^2^ = 38%; grade 3–4: RR = 0.61, 95% CI: 0.48–0.77, *P* < 0.01, I^2^ = 0%). Heterogeneity between olaparib group and control groups improved after further exclusion of 2 studies [18, 19] with olaparib 800 mg/day (any grade: 22%; grade 3–4: 0%). The results of our meta-analysis showed that olaparib 800 mg/day (without olaparib plus cediranib regime) may be associated with a higher risk of hypertension (any grade: RR = 2.71, 95% CI: 1.10–6.69, *P* = 0.03, I^2^ = 23%). However, olaparib 200 mg-600 mg/day(exclude olaparib plus cediranib regime) was associated with a lower risk of hypertension compared with the control group (any grade: RR = 0.79, 95% CI: 0.69–0.89, *P* < 0.01, I^2^ = 22%; grade 3–4: RR = 0.61, 95% CI: 0.48–0.77, *P* < 0.01, I^2^ = 0%). See Table 3 and Supplementary Fig. 3 for details.

#### Subgroup analysis of cancer type

Based on the cancer type, we performed a subgroup analysis of five cancers including ovarian cancer, lung cancer, breast cancer, prostate cancer, and pancreatic cancer. All five subgroups showed no statistically significant difference in the risk of hypertension between the PARP inhibitor group and the control group. See Table 3 and Supplementary Fig. 4 for details. Other cancers were not analyzed separately because too few studies were included.

#### Subgroup analysis of treatment regime

Based on treatment regime, we divided the study into three subgroups: PARP inhibitors versus placebo, PARP inhibitors versus other anticancer drugs, PARP inhibitors + other anticancer drugs versus other anticancer drugs, and All three subgroups showed no statistically significant difference in the risk of hypertension between the PARP inhibitors group and the control group. See Table 3 and Supplementary Fig. 5 for details.

### Publication Bias

For studies reporting hypertension of any grade and grades 3–4, neither the corresponding funnel plot nor Begg's test values indicated significant publication bias. See Supplementary Fig. 6 and Supplementary Table 2 for details.

## Discussion

PARP inhibitors have shown good clinical efficacy in clinical trials, especially in BRCA-mutant ovarian cancer and breast cancer, but accompanied by some adverse events. At present, the systematic analysis of PARP inhibitor related adverse events mainly involves gastrointestinal adverse events [50], hematological adverse events [51], pneumonitis [52], myelodysplastic syndrome and acute myeloid leukaemia [53], peripheral neuropathy [54], etc. However, there is no comprehensive and systematic analysis of PARP inhibitor-related hypertension, although many clinical trials have reported different grades and proportions of PARP inhibitor-related hypertension. This is the first meta-analysis to systematically assess the incidence and risk of PARP inhibitor-related hypertension in cancer patients. We conducted a meta-analysis of 32 phase II or III RCTs involving 10,654 participants, and further analyzed the incidence of hypertension with different PARP inhibitors, as well as the risk of hypertension with different PARP inhibitors, different cancer types, and different treatment regimes. The results of our analysis involved olaparib, veliparib, niraparib, rucaparib and iniparib 5 PARP inhibitors.

Gastrointestinal and hematological adverse events are the most common adverse events of PARP inhibitors. The incidence of any grade hypertension associated with PARP inhibitors was 12%, which was lower than any grade gastrointestinal (nausea: 68.8%, vomiting: 47.8%, diarrhea: 25.3%, constipation: 25.3%) and hematological (anemia:47.8%, neutropenia: 39.6%, thrombocytopenia:23.0%) adverse events associated with PARP inhibitors [50, 51]. The incidence of grade 3–4 hypertension related to PARP inhibitors is 4%, which is higher than grade 3–4 gastrointestinal toxicity (nausea: 3.4%, vomiting: 2.0%, diarrhea: 1.7% and constipation: 1.4%) related to PARP inhibitors and lower than grade 3–4 hematological toxicity (anemia: 22.1%, neutropenia: 19.3%, thrombocytopenia: 15.4%) related to PARP inhibitors [50, 51]. There is a great difference in the incidence of hypertension among PARP inhibitors. Olaparib (any grade: 17%, grades 3–4: 7%) and niraparib (any grade: 16%, grades 3–4: 5%) all show high incidence of hypertension, and their incidence of grade 3–4 hypertension is similar to that of sorafenib [55] (5.7%, a tyrosine kinase inhibitors). However, the incidence of hypertension in veliparib (any grade: 8%, grade3-4: 1%) and rucaparib (any grade: 6%, grade3-4: 2%) is not high. PARP inhibitor-related hypertension may be due to an off target disruption of dopamine and nor epinephrine metabolism [56].

The results of our meta-analysis showed no statistically significant difference in the risk of hypertension between the PARP inhibitor group and the control group, but this result is not completely reliable because of large heterogeneity among studies (any grade: I^2^ = 80%, grade 3–4: I^2^ = 68%). Our subgroup analyses of cancer types and treatment regimes were consistent with the results of total PARP inhibitors, but there was also substantial heterogeneity across studies. Finally, we found that the varieties of PARP inhibitors maybe the main source of heterogeneity, and the risk of hypertension varied widely among different PARP inhibitors. Niraparib exhibited a significantly higher risk of hypertension than the control group. Niraparib-related hypertension may be attributable to off-target disruption of dopamine and norepinephrine metabolism and inhibition of DYRK1A (dual-specificity tyrosine phosphorylated and regulated kinase 1A) [57]. However, the risk of hypertension with veliparib and rucaparib was similar to the control group, and olaparib may even reduced the risk of hypertension in some cases.

Olaparib is currently the most widely investigated PARP inhibitor and has demonstrated promising efficacy in various cancers such as ovarian cancer and breast cancer [58, 59]. Our analysis of olaparib is very interesting. On the one hand, the risk of hypertension with olaparib was associated with combination therapy. The risk of hypertension was significantly higher in the olaparib plus cediranib regimen than in the control group, whereas olaparib alone or in combination with other anticancer drugs showed the opposite results. The results of the olaparib plus cediranib regimen was consistent with the meta-analysis of Guo et al. [60], which may be mainly attributed to the inhibition of vascular endothelial growth factor receptor by cediranib [61], or some mechanism of the combination of the two drugs. On the other hand, the risk of hypertension with olaparib is dose-related. Olaparib (without olaparib plus cediranib regimen) 800 mg/day having a significantly higher risk of hypertension than the control group, while olaparib200mg-600 mg/day(exclude olaparib plus cediranib regimen) had a lower risk of hypertension than the control group. One study [62] found that PARP inhibitors may have an inhibitory effect on angiotensin II (Ang II) in rats, so we speculate that olaparib may reduces the risk of hypertension by inhibiting renin angiotensin system (RAS), an important factor in the occurrence and maintenance of essential hypertension [63]. The mechanism of olaparib 800 mg/day increasing the risk of hypertension is unclear, and further research is needed.

Our previous results show that olaparib has a high incidence of hypertension, which seems to contradict the result that olaparib reduces the risk of hypertension. So, we excluded the study involving olaparib 800 mg/day and olaparib plus cediranib regimen and calculated the incidence of hypertension in the olaparib and control groups, respectively. The results showed that the incidence of hypertension in oalparib group (any grade: 11%, grade 3–4: 3%) was lower than that in the control group(any grade: 15%, grade 3–4: 5%), which was consistent with the result that olaparib reduced the risk of hypertension (Supplementary Fig. 3E and Supplementary Fig. 3F).

For patients receiving niraparib, olaparib 800 mg/day and the combination of olaparib and cediranib, some measures should be taken to prevent the development of hypertension, such as limiting salt intake (< 5 g/day), regular aerobic exercise supplemented by dynamic resistance exercise and flexible exercise, etc. [64, 65]. At the same time, clinicians should monitor and control patients' blood pressure and give medication in severe cases. According to relevant guidelines [66, 67], angiotensin-converting enzyme inhibitors (ACEI) or angiotensin receptor blockers (ARB), beta receptor blockers, diuretics and calcium channel blockers (CCB) are currently the mainstream drugs for the treatment of hypertension. Clinicians can select appropriate drugs to treat cancer patients with PARP inhibitor-associated hypertension.

This meta-analysis has five limitations. First of all, there is a lack of relevant single-arm studies when assessing the incidence of hypertension associated with PARP inhibitors. Second, more than one-third of RCTs are open-label studies that are not blinded. Thirdly, the duration of treatment, duration of follow-up, and median age varied widely among the included studies. Fourthly, we only retrieved one eligible study involving iniparib, and not any eligible studies involving pamiparib, fuzuloparib and talazoparib, because the relevant clinical studies were mainly concentrated in phase I. Finally, Because there are too few relevant studies, we did not compare the risk of hypertension between different doses of olaparib in cancer patients.

## Conclusion

The incidence and risk of hypertension varied widely among different PARP inhibitors. Olaparib 200-600 mg/day (excluding olaparib plus cediranib regimen) may be the most suitable PARP inhibitor for cancer patients with high risk of hypertension, followed by veliparib and rucaparib. Niraparib, olaparib 800 mg/day, and the combination of olaparib and cediranib all have a high risk of hypertension. Therefore, cancer patients who use the above drugs should strengthen blood pressure monitoring and take some simple preventive measures, and receive appropriate medication in severe cases.

## Supplementary Information

**Additional file 1: Supplementary Table 1.** Quality evaluation of RCTs according to Cochrane Collaboration Guidelines. **Supplementary Figure 1.** Risk of total PARP inhibitor-related hypertension. **Supplementary Figure 2.** Risk of hypertension with different PARP inhibitors. **Supplementary Figure 3.** Detailed analysis of olaparib-related hypertension. **Supplementary Figure 4.** Risk of hypertension in different types of cancer. **Supplementary Figure 5.** Risk of hypertension in different treatment regime. **Supplementary Table 2.** Begg's test results of any grade and grade 3-4 hypertension related to total PARP inhibitor. **Supplementary Figure 6.** Funnel plot of hypertension associated with total PARP inhibitors.

## Data Availability

The datasets used and/or analyzed during the current study are available from the corresponding author on reasonable request.
