# Supplementary material for: Incidence and risk of hypertension associated with PARP inhibitors in cancer patients: a systematic review and meta-analysis
Source: BMC Cancer. 2023 Jan 31;23:107. doi: 10.1186/s12885-023-10571-5 (PMC9887889; doi:10.1186/s12885-023-10571-5)
Supplement: Supplementary file 1 — Additional file 1: Supplementary Table 1. Quality evaluation of RCTs according to Cochrane Collaboration Guidelines. Supplementary Figure 1. Risk of total PARP inhibitor-related hypertension. Supplementary Figure 2. Risk of hypertension with different PARP inhibitors. Supplementary Figure 3. Detailed analysis of olaparib-related hypertension. Supplementary Figure 4. Risk of hypertension in different types of cancer. Supplementary Figure 5. Risk of hypertension in different treatment regime. Supplementary Table 2. Begg's test results of any grade and grade 3-4 hypertension related to total PARP inhibitor. Supplementary Figure 6. Funnel plot of hypertension associated with total PARP inhibitors. [file 12885_2023_10571_MOESM1_ESM.docx]

**Supplementary information for**

**Incidence and risk of hypertension associated with PARP inhibitors in cancer patients: a systematic review and meta-analysis**

**1.Supplementary Table 1:** Quality evaluation of RCTs according to Cochrane Collaboration Guidelines.

**2. Supplementary Figure 1.** Risk of total PARP inhibitor-related hypertension.

**3. Supplementary Figure 2.** Risk of hypertension with different PARP inhibitors.

**4. Supplementary Figure 3.** Detailed analysis of olaparib-related hypertension.

**5. Supplementary Figure 4.** Risk of hypertension in different types of cancer.

**6. Supplementary Figure 5.** Risk of hypertension in different treatment regime.

**7.** **Supplementary Table 2.** Begg's test results of any grade and grade 3-4 hypertension related to total PARP inhibitor.

**8**. **Supplementary Figure 6.** Funnel plot of hypertension associated with total PARP inhibitors.

**1.** **Supplementary Table 1:** Quality evaluation of RCTs according to Cochrane Collaboration Guidelines.

| Study | A | B | C | D | E | F | G | Overall |
| --- | --- | --- | --- | --- | --- | --- | --- | --- |
| Kaye et.al 2011 | low | high | high | high | high | low | low | high |
| Novello et.al 2014 | unclear | high | high | high | high | unclear | low | high |
| Han et.al 2018 | low | unclear | low | low | high | low | low | low |
| Hussain et.al 2017 | unclear | high | high | high | low | low | low | high |
| Owonikoko et.al 2020 | unclear | unclear | unclear | unclear | low | unclear | unclear | unclear |
| Banerjee et.al 2021 | low | low | low | low | low | low | low | low |
| Mirza et.al 2016 | unclear | unclear | low | low | low | unclear | low | unclear |
| Bang et.al 2017 | low | low | low | low | low | low | low | low |
| Ledermann et.al 2021 | low | low | low | low | low | low | high | low |
| Loibl et.al 2018 | low | low | low | low | low | low | low | low |
| Gorbunova et.al 2019 | low | unclear | low | low | unclear | low | low | low |
| Ray‑Coquard et.al 2019 | low | unclear | unclear | unclear | low | unclear | high | unclear |
| González‑Martín et.al 2019 | low | low | low | low | low | low | low | low |
| Bono et.al 2020 | low | high | high | low | low | low | low | low |
| Ai et.al 2021 | low | low | low | low | high | low | unclear | low |
| Wu et.al 2020 | low | low | low | low | low | low | unclear | low |
| Sun et.al 2022 | low | unclear | unclear | unclear | high | unclear | high | high |
| Liu et.al 2021 | low | high | high | high | low | low | low | high |
| Woll et.al 2022 | low | low | low | low | low | low | low | low |
| O’Reilly et.al 2020 | unclear | high | high | high | low | unclear | unclear | high |
| Pusztai et.al 2021 | low | high | high | high | unclear | low | low | high |
| Ledermann et.al 2012 | low | low | low | low | low | low | low | low |
| Kristeleit et.al 2022 | low | high | high | high | low | low | low | low |
| Colombo et.al 2022 | low | high | high | high | low | unclear | unclear | low |
| Diéras et.al.2020 | low | low | low | high | low | low | low | low |
| Chiorean et.al 2021 | low | high | high | high | low | low | low | high |
| Golan et.al 2020 | low | low | low | low | unclear | low | low | low |
| Coleman et.al 2019 | low | high | low | high | low | low | low | low |
| Clarke et.al 2018 | low | low | low | low | low | low | low | low |
| Rugo et.al 2016 | low | high | high | high | low | low | low | high |
| Monk et.al 2022 | low | low | low | low | low | unclear | unclear | unclear |
| Fennell et.al 2022 | low | low | low | unclear | low | unclear | unclear | low |

A: Random sequence generation; B:Allocation concealment; C: Blinding of participants and personnel; D: Blinding of outcome assessment; E: Incomplete outcome data; F: Selective reporting; G: Other sources of bias.

**2. Supplementary Figure 1.** Risk of total PARP inhibitor-related hypertension.

**
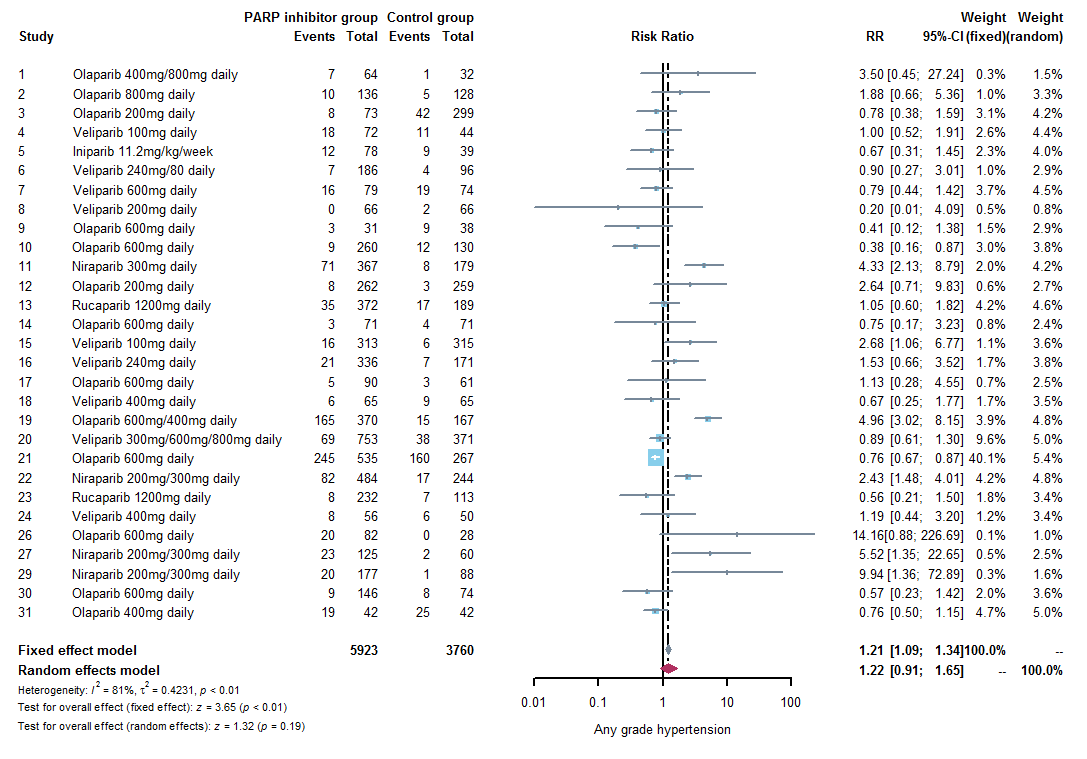
**

**Figure 1A.** Risk of any grade hypertension with total PARP inhibitors. PARP: poly(adenosine diphosphate-ribose) polymerase; RR: risk ratio; 95%CI: 95% confidence intervals. If I^2^< 50%, the fixed effect model is used for analysis, otherwise, the random effect model is used for analysis.

**
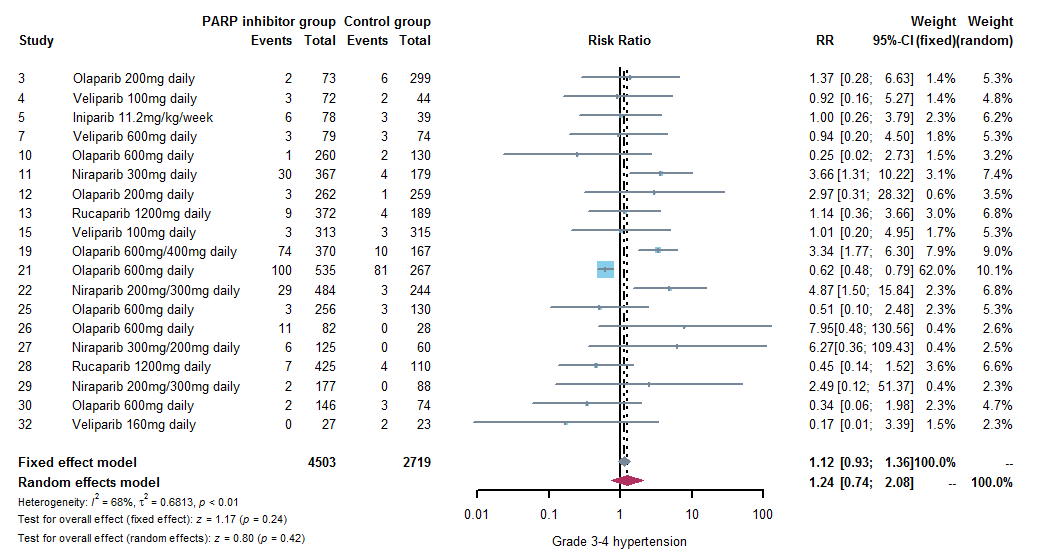
**

**Figure 1B.** Risk of grade 3-4 hypertension with total PARP inhibitors. PARP: poly(adenosine diphosphate-ribose) polymerase; RR: risk ratio; 95%CI: 95% confidence intervals. If I^2^< 50%, the fixed effect model is used for analysis, otherwise, the random effect model is used for analysis.

**3. Supplementary Figure 2.** Risk of hypertension with different PARP inhibitors.


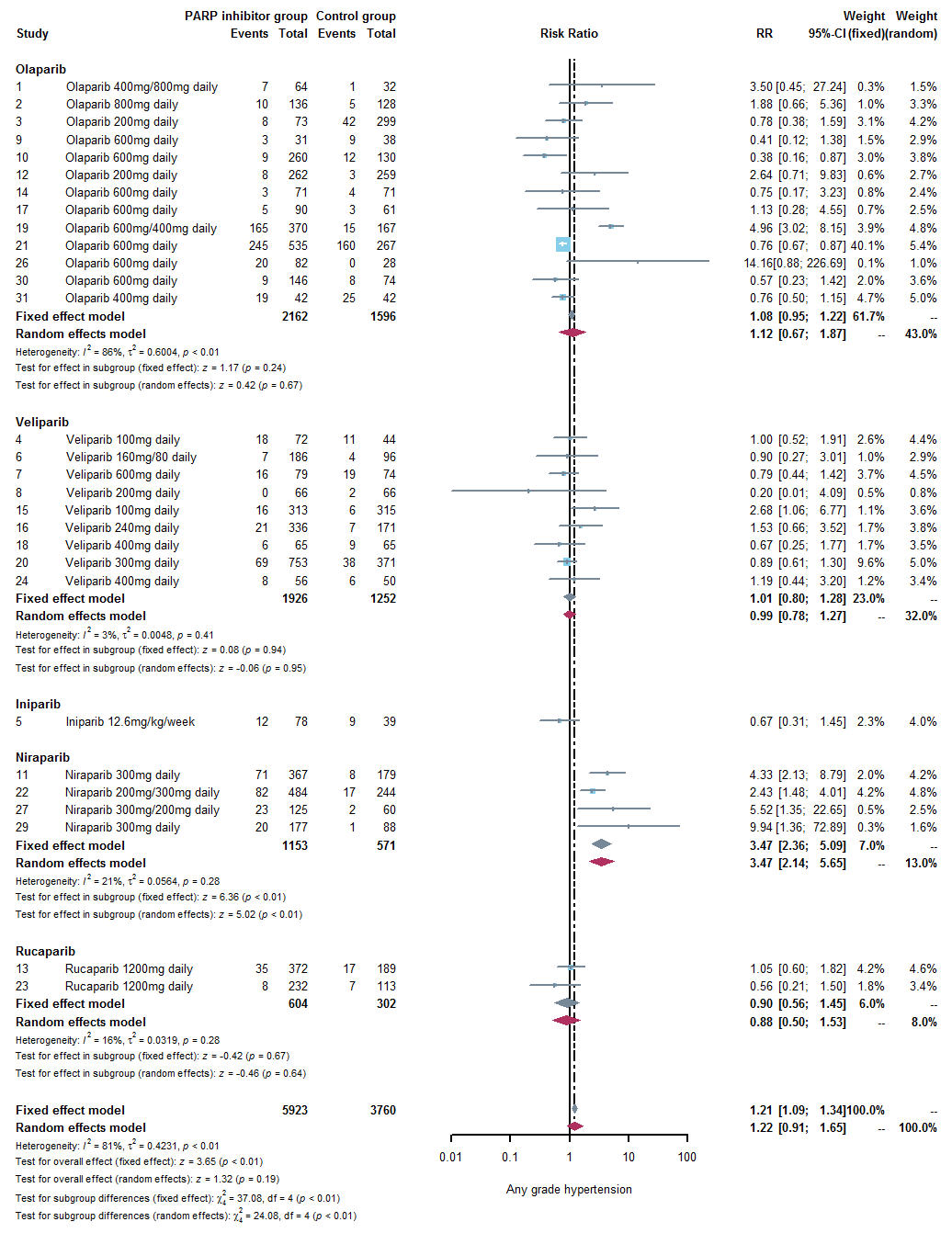


**Figure 2A.** Risk of any grade hypertension with different PARP inhibitors. PARP: poly(adenosine diphosphate-ribose) polymerase; RR: risk ratio; 95%CI: 95% confidence intervals. If I^2^< 50%, the fixed effect model is used for analysis, otherwise, the random effect model is used for analysis.


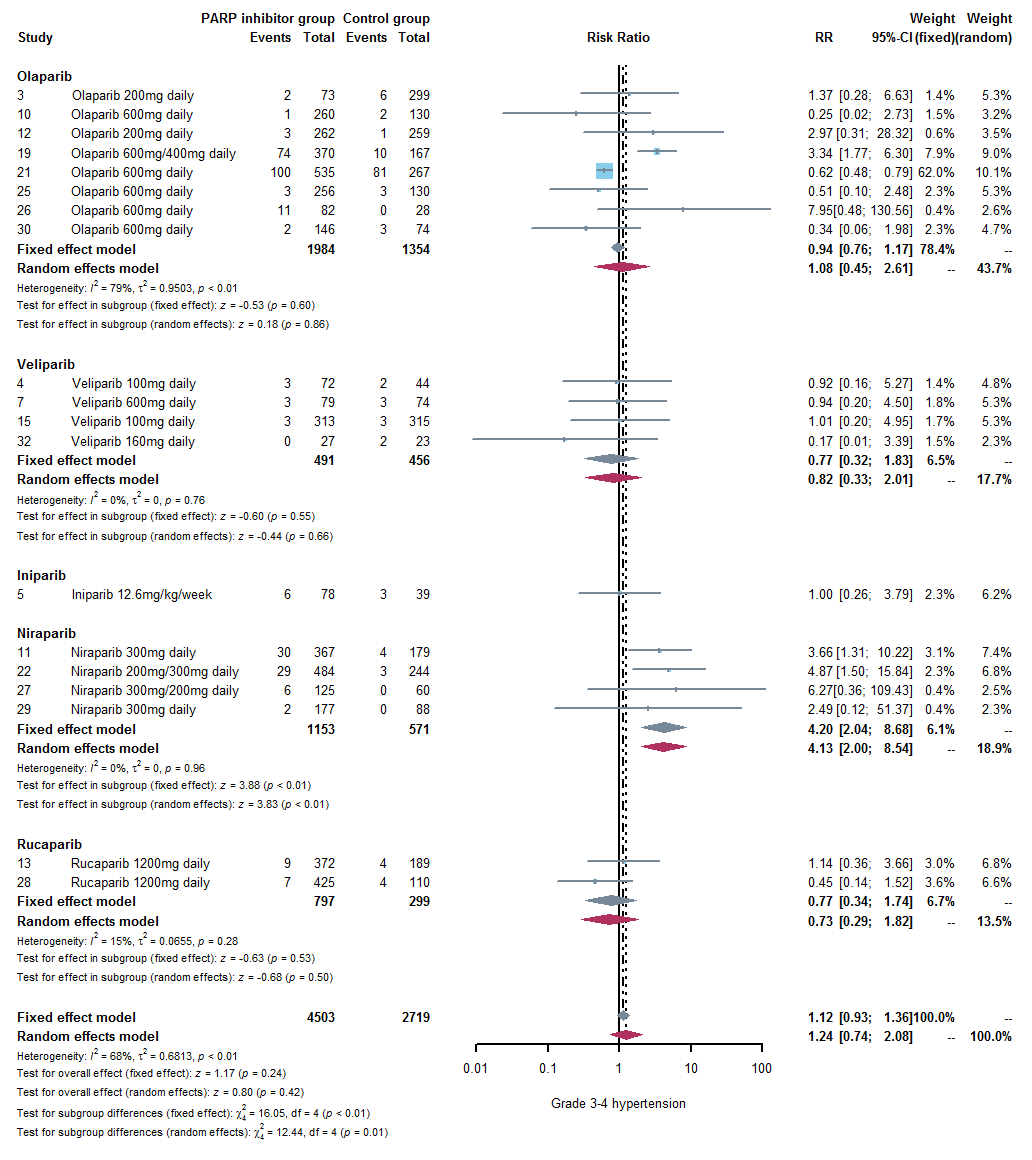


**Figure 2B.** Risk of grade 3-4 hypertension with different PARP inhibitors. PARP: poly(adenosine diphosphate-ribose) polymerase; RR: risk ratio; 95%CI: 95% confidence intervals. If I^2^< 50%, the fixed effect model is used for analysis, otherwise, the random effect model is used for analysis.

**4. Supplementary Figure 3.** Detailed analysis of olaparib-related hypertension.


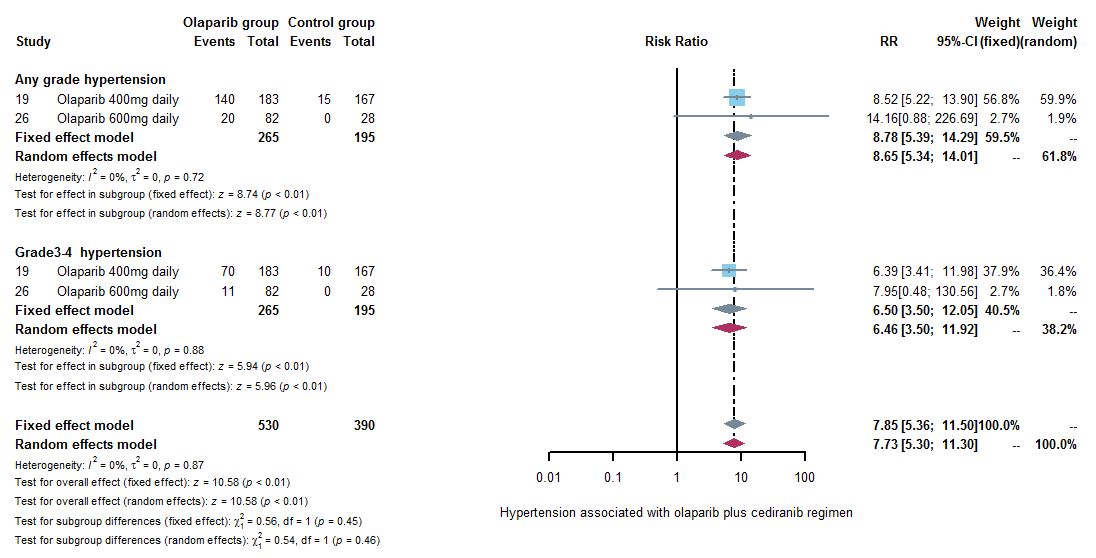


**Figure 3A.** Any grade and grade 3-4 hypertension risk of olaparib combined with cediranib. RR: risk ratio; 95%CI: 95% confidence intervals. If I^2^< 50%, the fixed effect model is used for analysis, otherwise, the random effect model is used for analysis.


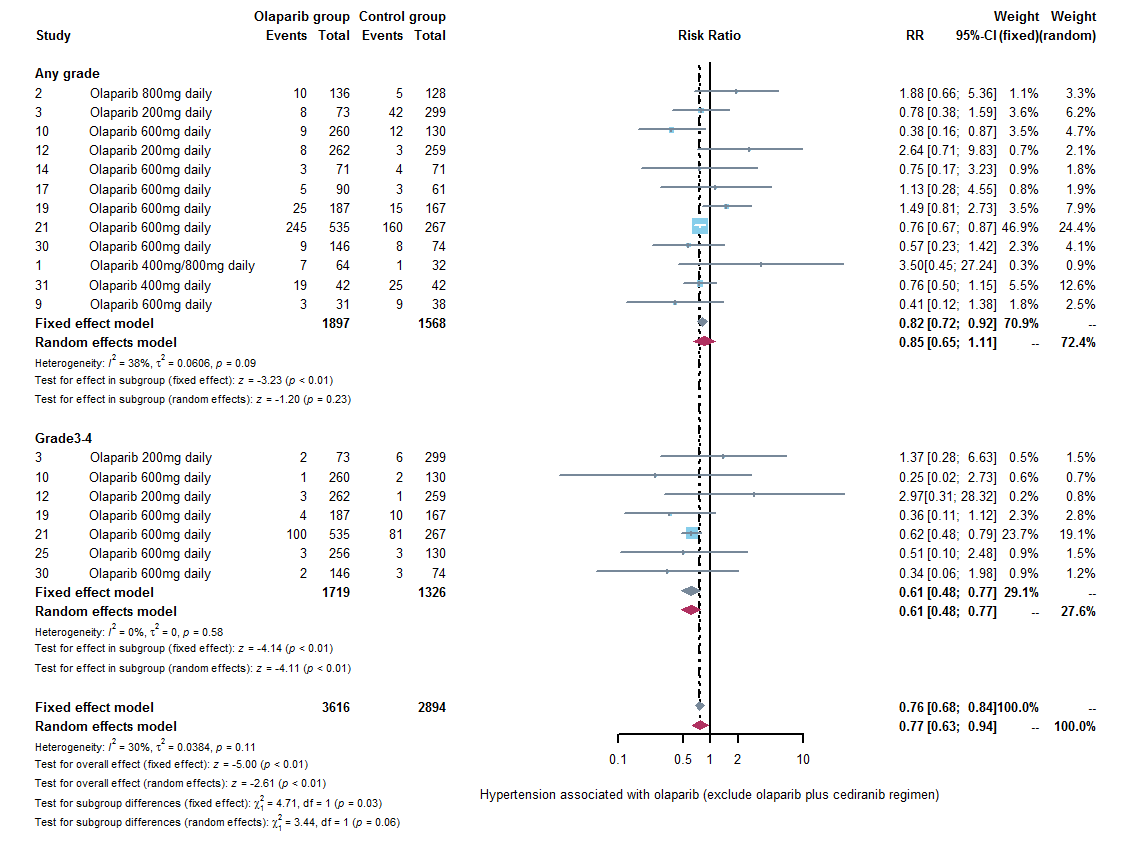


**Figure 3B.** Any grade and grade 3-4 hypertension risk of olaparib ( exclude olaparib plus cediranib regime). RR: risk ratio; 95%CI: 95% confidence intervals. If I^2^< 50%, the fixed effect model is used for analysis, otherwise, the random effect model is used for analysis.


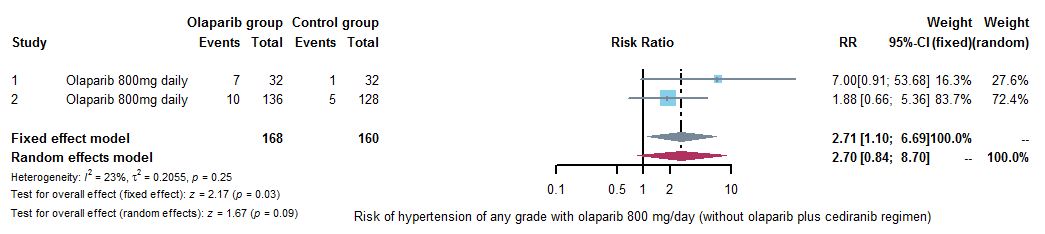


**Figure 3C.** Risk of hypertension of any grade with olaparib 800 mg daily (without olaparib plus cediranib regimen). RR: risk ratio; 95%CI: 95% confidence intervals. If I^2^< 50%, the fixed effect model is used for analysis, otherwise, the random effect model is used for analysis.


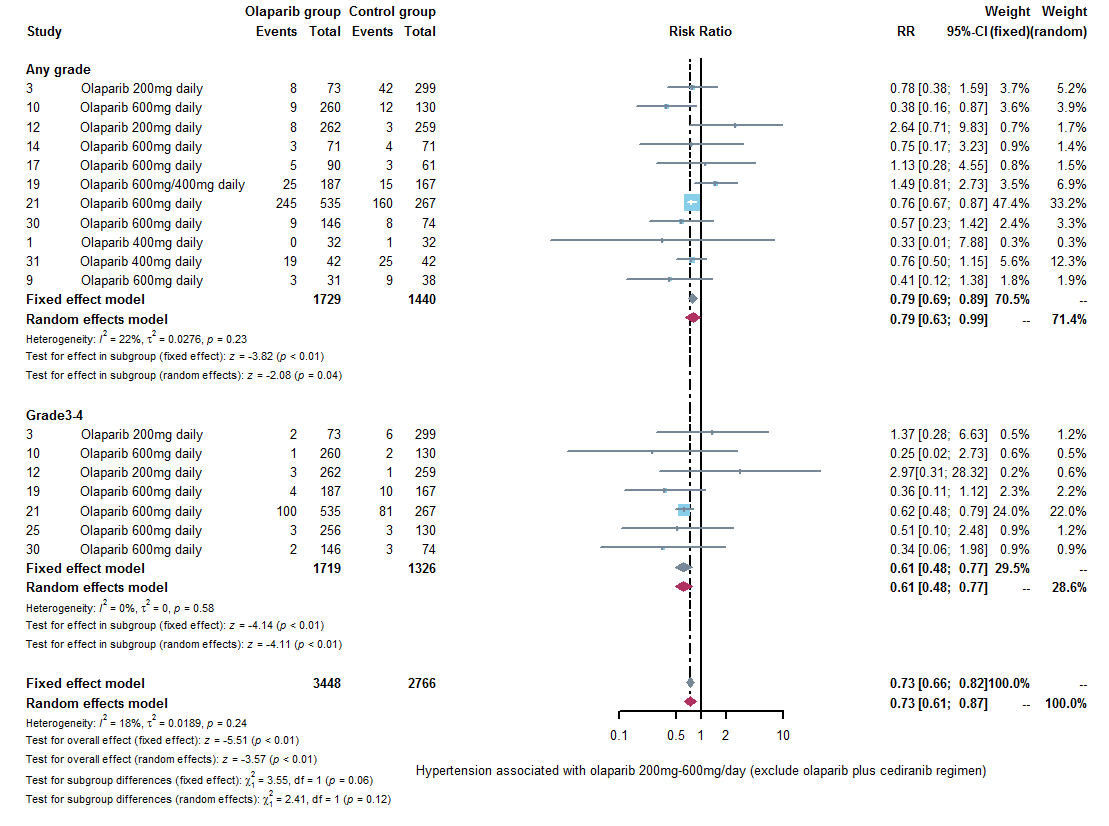


**Figure 3D.** Any grade and grade 3-4 hypertension risk of olaparib 200mg-600mg/day (exclude olaparib plus cediranib regime). RR: risk ratio; 95%CI: 95% confidence intervals. If I^2^< 50%, the fixed effect model is used for analysis, otherwise, the random effect model is used for analysis.


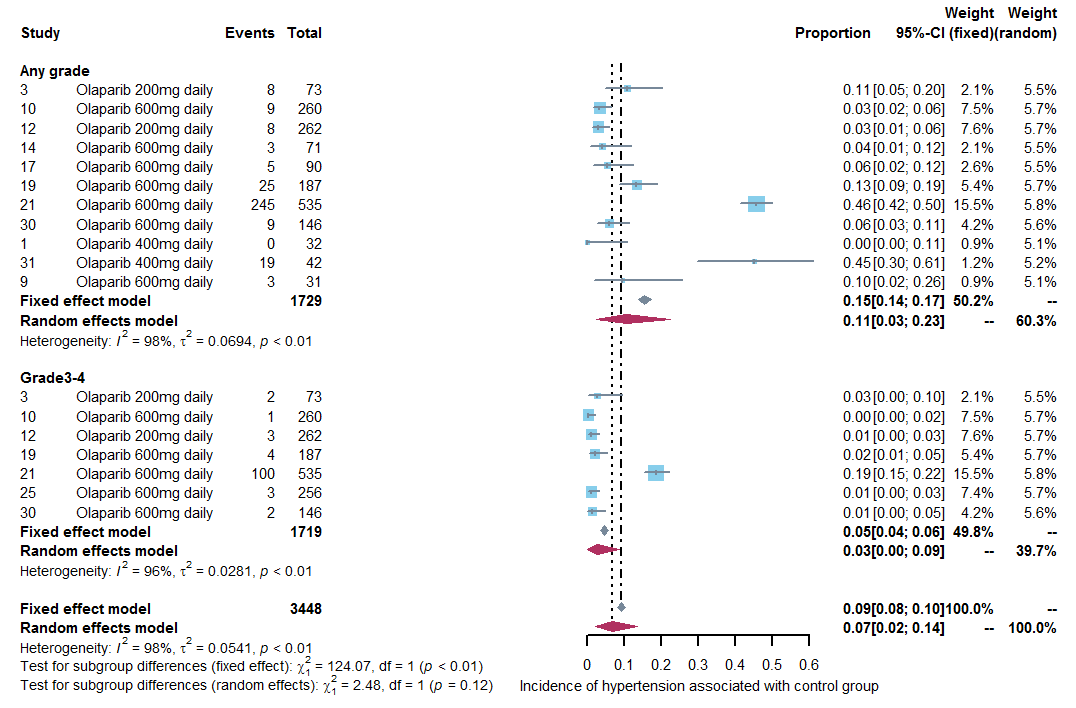


**Figure 3E.** Incidence of hypertension associated with olaparib 200mg-600mg/day group (excluding olaparib plus cediranib regimen). 95%CI: 95% confidence intervals. If I^2^< 50%, the fixed effect model is used for analysis, otherwise, the random effect model is used for analysis.


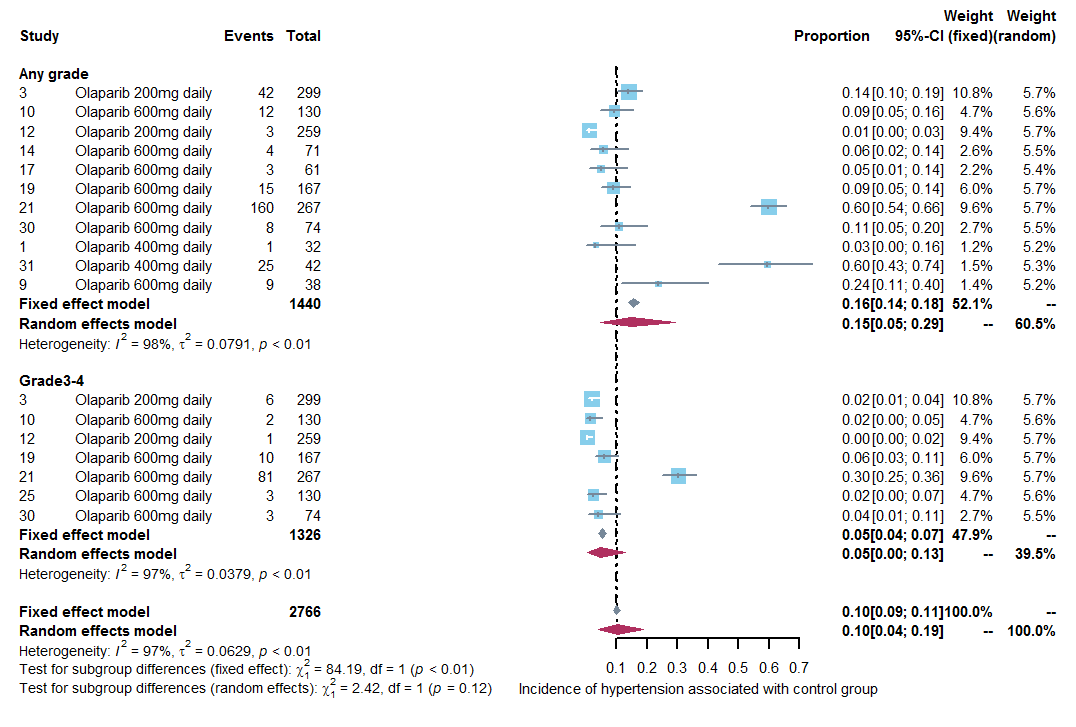


**Figure 3F.** Incidence of hypertension associated with control group(excluding olaparib plus cediranib regimen). 95%CI: 95% confidence intervals. If I^2^< 50%, the fixed effect model is used for analysis, otherwise, the random effect model is used for analysis.

**5. Supplementary Figure 4.** Risk of hypertension with different cancer types.

**
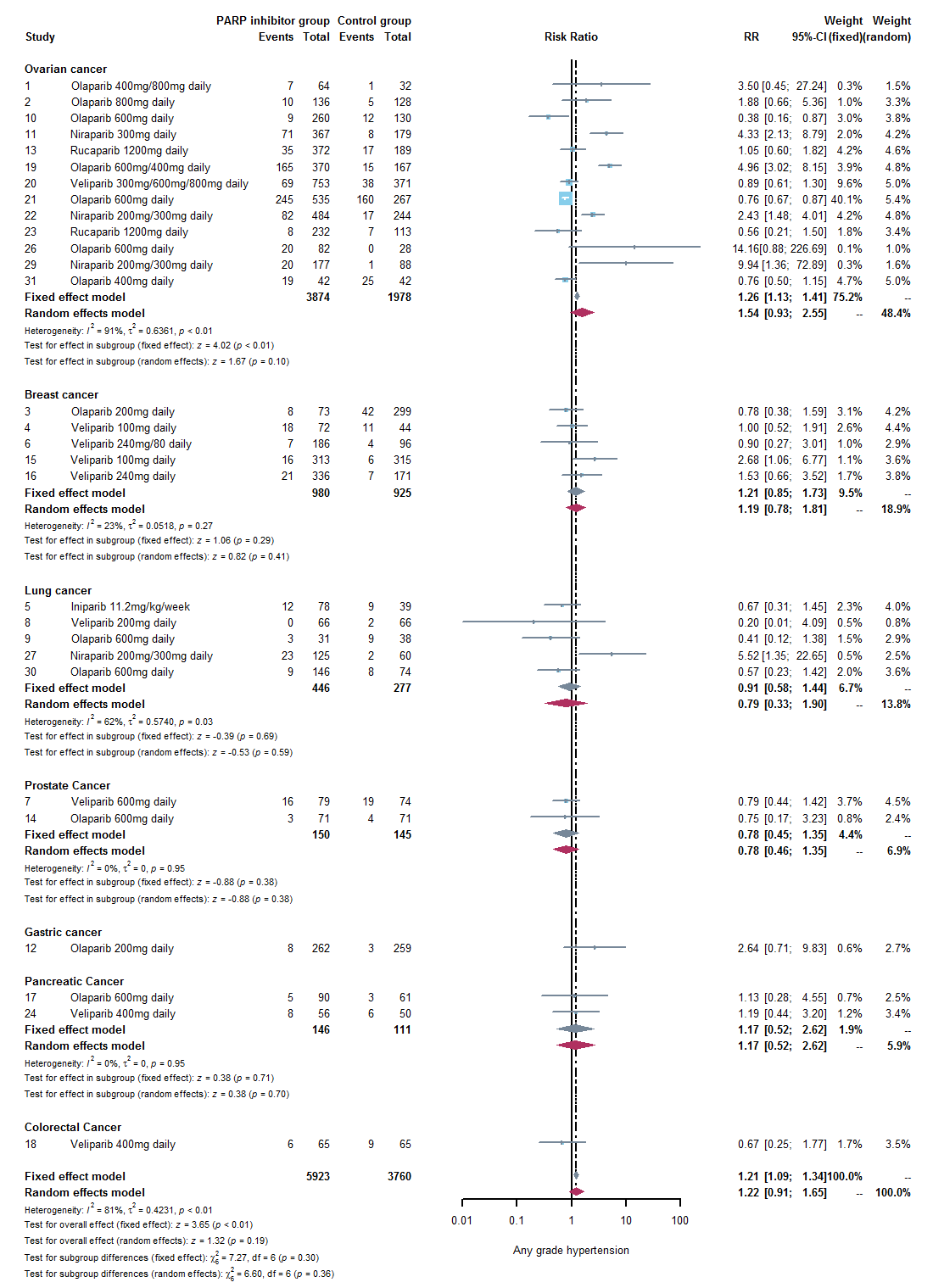
**

**Figure 4A.** Risk of any grade hypertension with different cancer types. PARP: poly(adenosine diphosphate-ribose) polymerase; RR: risk ratio; 95%CI: 95% confidence intervals. If I^2^< 50%, the fixed effect model is used for analysis, otherwise, the random effect model is used for analysis.

**
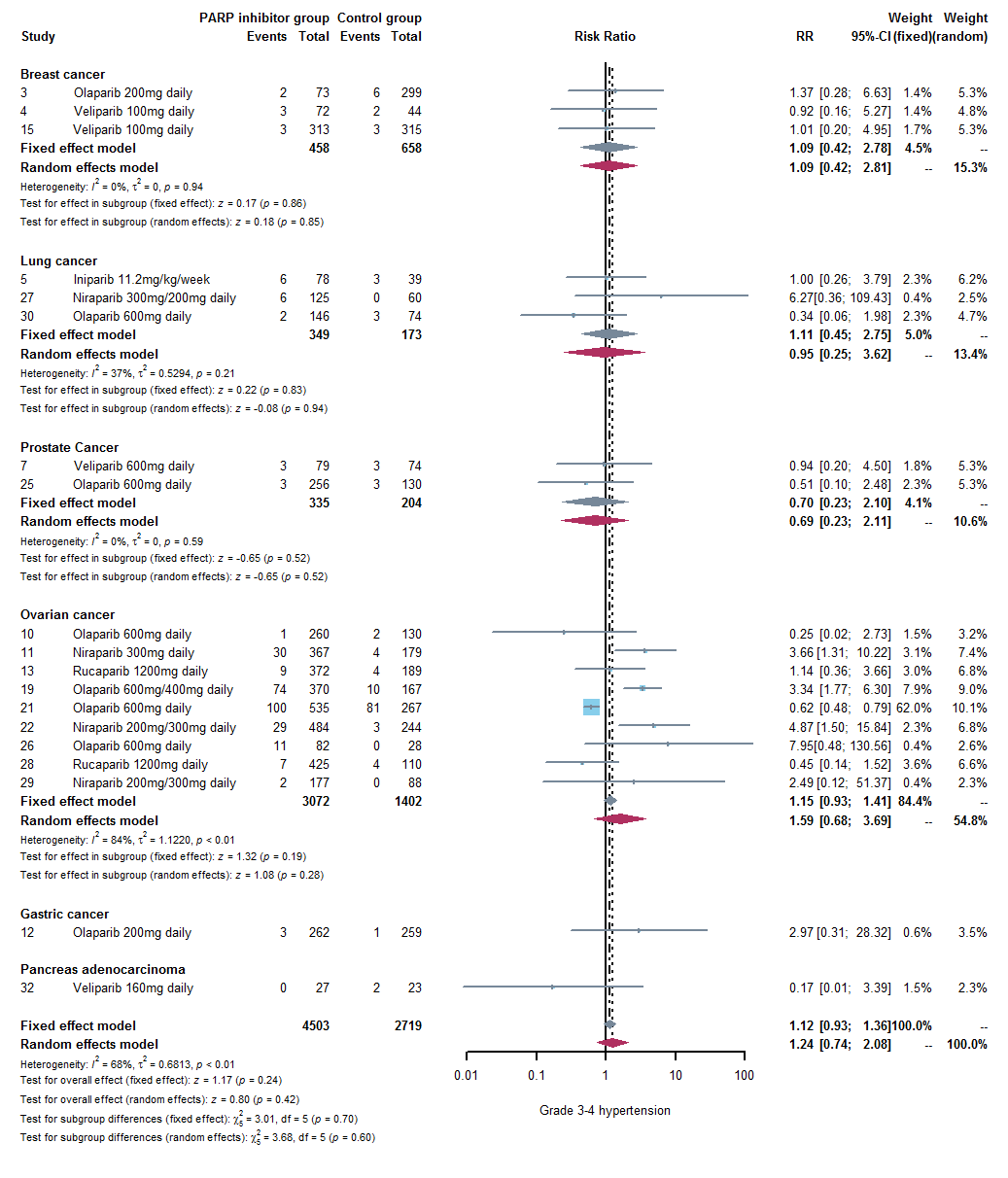
**

**Figure 4B.** Risk of grade 3-4 hypertension with different PARP inhibitors. PARP: poly(adenosine diphosphate-ribose) polymerase; RR: risk ratio; 95%CI: 95% confidence intervals. If I^2^< 50%, the fixed effect model is used for analysis, otherwise, the random effect model is used for analysis.

**6. Supplementary Figure 5.** Risk of hypertension in different treatment regimes.

**
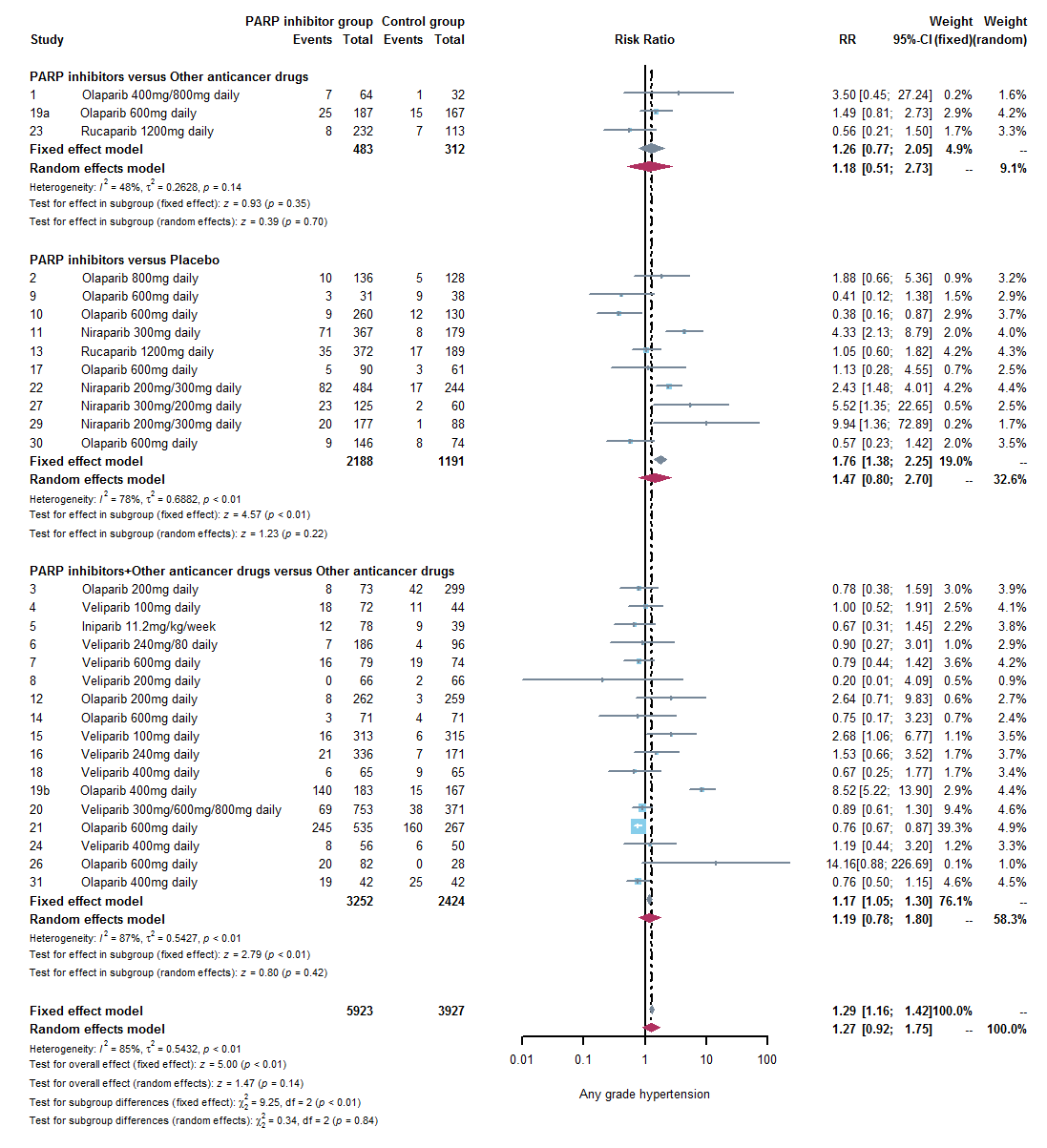
**

**Figure 5A.**Risk of any grade hypertension with different treatment regimes. PARP: poly(adenosine diphosphate-ribose) polymerase; RR: risk ratio; 95%CI: 95% confidence intervals. If I^2^< 50%, the fixed effect model is used for analysis, otherwise, the random effect model is used for analysis.

**
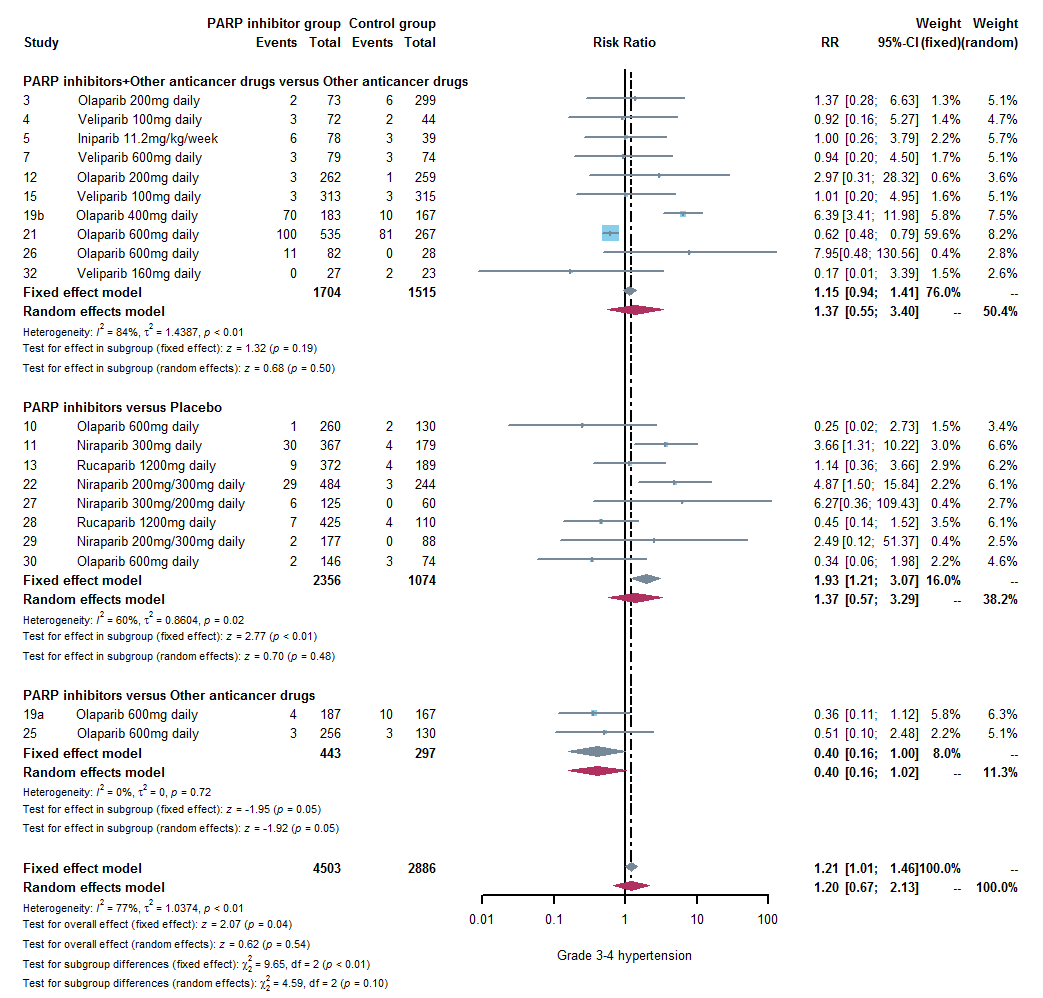
**

**Figure 5B.**Risk of grade 3-4 hypertension with different treatment regimes. PARP: poly(adenosine diphosphate-ribose) polymerase; RR: risk ratio; 95%CI: 95% confidence intervals. If I^2^< 50%, the fixed effect model is used for analysis, otherwise, the random effect model is used for analysis.

**7.** **Supplementary Table 2:**Begg's test results of any grade and grade 3-4 hypertension related to total PARP inhibitor.

|  | P value (any grade) | P value (grade 3-4) |
| --- | --- | --- |
| PARP inhibitors | 0.4308 | 0.5997 |

PARP: Poly-ADP ribose polymerase. If *P*<0.05, it indicates that there is publication bias.

**8**. **Supplementary Figure 6.** Funnel plot of hypertension associated with total PARP inhibitors.


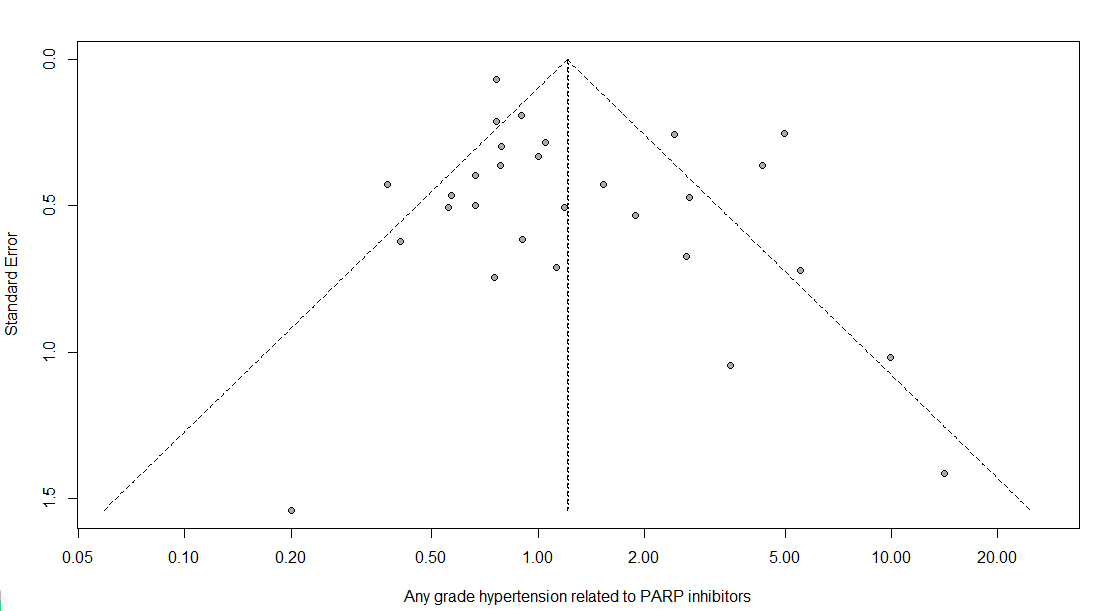


**Figure 6A.** Funnel plot of any grade hypertension related to total PARP inhibitor. PARP: poly(adenosine diphosphate-ribose) polymerase.


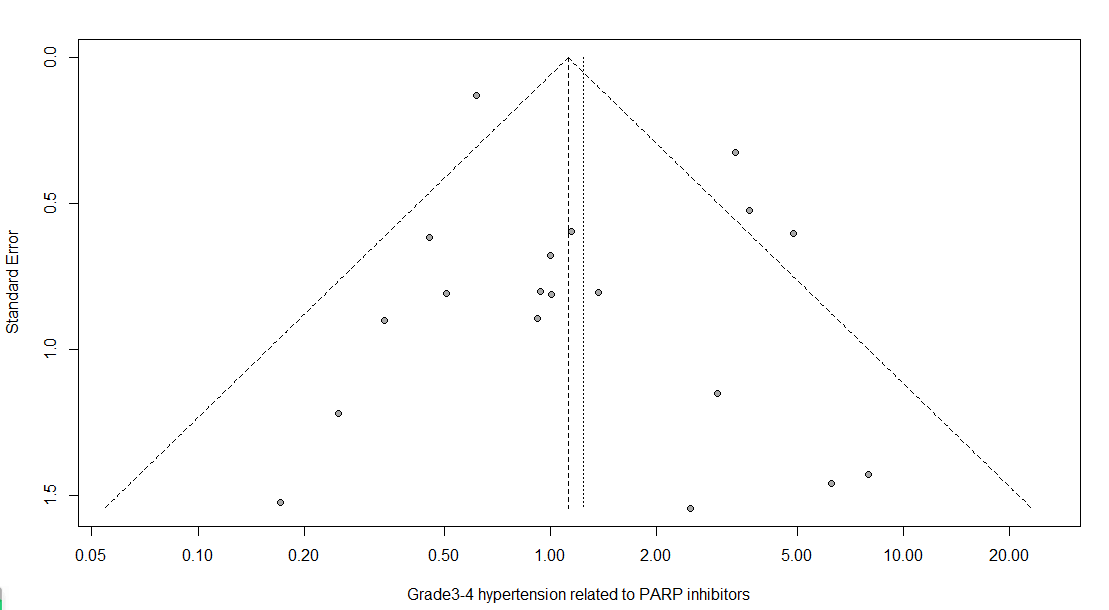


**Figure 6B.** Funnel plot of grade 3-4 hypertension related to total PARP inhibitor. PARP: poly(adenosine diphosphate-ribose) polymerase.
